# Prevalence of metabolic syndrome in patients with androgenetic alopecia: an analysis of current evidence

**DOI:** 10.3389/fendo.2026.1904726

**Published:** 2026-09-22

**Authors:** Wan Xu, Wen-qing Zhang, Xin Liang, Qian Fan, Hai-dong Yu, Xin Li

**Affiliations:** 1 Chinese Medicine Department, Songnan Town Community Health Service Center, Shanghai, China; 2 Department of Dermatology, Yueyang Hospital of Integrated Traditional Chinese and Western Medicine, Shanghai University of Traditional Chinese Medicine, Shanghai, China; 3 Institute of Dermatology, Shanghai Academy of Traditional Chinese Medicine, Shanghai, China

**Keywords:** androgenic alopecia, meta-analysis, metabolic syndrome, prevalence rate, systematic review

## Abstract

**Background:**

Androgenetic alopecia (AGA) is the most common non-scarring alopecia, and its prevalence increases with age. Previous meta-analyses of AGA and metabolic syndrome (MetS) have focused mainly on associations and risk estimates. We quantified the prevalence of MetS and its components among patients with AGA.

**Methods:**

PubMed, Embase, Web of Science, the Cochrane Library, CNKI, VIP, and WanFang Data were searched from inception to July 2026 for cross-sectional and cohort studies. Prevalence estimates were pooled using logit-transformed random-effects models with restricted maximum likelihood estimation. We report 95% confidence intervals (CIs), I², τ², and 95% prediction intervals (PIs) when estimable. Examined subgroups included region, sex, AGA severity, MetS definition, and study quality. Sensitivity analyses assessed robustness. Egger’s test was performed to assess outcomes derived from ≥10 studies.

**Results:**

Twenty-eight studies were included. MetS prevalence was 23.7% (12 studies; 3,623 participants; 95% CI, 15.5%–34.5%; I² = 95.4%; 95% PI, 3.7%–71.7%). Prevalence estimates for abdominal obesity, hypertension or elevated blood pressure, diabetes mellitus, dyslipidemia, hypertriglyceridemia, elevated low-density lipoprotein cholesterol, and low high-density lipoprotein cholesterol were 36.0%, 30.7%, 13.3%, 24.5%, 23.6%, 30.0%, and 28.8%, respectively. Heterogeneity was substantial across outcomes; the elevated low-density lipoprotein cholesterol estimate was based on only two studies. MetS prevalence was 19.7%, 30.8%, and 44.2% in mild, moderate, and severe AGA, respectively, although grading systems varied. In leave-one-out analyses, the pooled MetS estimate ranged from 21.1% to 25.9%; other outcomes with at least seven studies were also broadly unchanged; however, heterogeneity persisted. Evidence from female patients was limited (4 studies, 765 participants).

**Conclusion:**

MetS and several metabolic abnormalities are common among patients with AGA, although estimates vary substantially across studies. Early assessment may support comprehensive health management in this population.

## 1 Introduction

Androgenetic alopecia (AGA) is a common chronic, non-scarring hair disorder characterized by progressive miniaturization of hair follicles (1). In men, it is referred to as male androgenetic alopecia (MAGA), whereas in women it is referred to as female androgenetic alopecia (FAGA) (1). MAGA often becomes apparent during the second decade of life, affects approximately 30% of men by age 30 and 50% by age 50, and generally progresses with age (2). An epidemiological study conducted in six cities across China reported that the overall prevalence of AGA in men was 21.3% (3), while a Mexican study revealed the prevalence of AGA to be 28.76% (4). Currently, the incidence of AGA varies substantially with age, race, and geographical region, and notable discrepancies exist in the study designs of relevant literature (5). AGA has been reported in association with several comorbidities, including coronary artery disease (CAD), type 2 diabetes mellitus (DM), hypertension, obesity, dyslipidemia, insulin resistance, benign prostatic hyperplasia, and prostate cancer (6–8).

Metabolic syndrome (MetS) refers to a cluster of metabolic abnormalities associated with insulin resistance and obesity (9). A previous meta-analysis estimated the mean prevalence of MetS at 31% (10). Definitions of MetS vary across guidelines, particularly with respect to thresholds for waist circumference, serum triglycerides, and cholesterol. Initially observed in high-income countries, the prevalence of MetS has continued to increase, driven in part by adverse health behaviors such as physical inactivity and unhealthy diets (11); an increasing trend has also been reported among children and adolescents (12). Cardiovascular risk factors are common among men with early-onset AGA (13), suggesting that early assessment of MetS and its components may be beneficial in this population.

Numerous studies have examined the relationship between AGA and MetS. Reported MetS prevalence ranges from 21.0% to 68.9% among women with AGA (14–16) and from 10.0% to 73.3% among men with AGA (13, 17). A meta-analysis conducted in China found no direct association between AGA and MetS (18), whereas other meta-analyses reported a higher likelihood of MetS among patients with AGA, with risk estimates 3.46- to 11.86-fold those of control participants (19, 20). AGA has also been proposed as an independent indicator of cardiovascular disease and MetS (21). Given these findings, we systematically synthesized the available evidence to estimate the prevalence of MetS and its components among patients with AGA and explored potential variation by region, sex, AGA severity, and other study characteristics.

## 2 Study design and reporting

This systematic review and meta-analysis was conducted and reported in accordance with the PRISMA statement (**Supplementary Table 1**). The protocol was registered in PROSPERO (CRD420251078654). Literature searches, eligibility assessment, quality appraisal, and data extraction were performed independently by two reviewers, with disagreements resolved through discussion with a third reviewer. All stages, including searching, eligibility assessment, quality appraisal, data extraction, and analysis, followed a prespecified protocol.

## 3 Data sources and search strategy

PubMed, Embase, Web of Science, the Cochrane Library, CNKI, VIP, and WanFang Data were searched from database inception to July 2026. The search strategy combined three concept groups: (1) androgenetic and androgenic alopecias, male pattern baldness, female pattern hair loss, and corresponding subject headings; (2) metabolic syndrome, hypertension, diabetes mellitus, hyperlipidemia, and abdominal obesity; and (3) cross-sectional studies, prevalence, and corresponding subject headings. Controlled vocabulary was combined with title and abstract terms. Reference lists of included studies and relevant reviews were also screened. Complete search strategies for all databases are provided in **Supplementary Tables 2**, **3**.

## 4 Eligibility criteria

Studies were eligible if they: (1) enrolled patients diagnosed with AGA, male pattern hair loss, or female pattern hair loss according to clinical or study-defined criteria; (2) used a cross-sectional or cohort design; (3) reported, or allowed unique derivation of, the numerator and denominator for an outcome of interest among patients with AGA; and (4) were available as full-text articles in Chinese or English. We excluded case-control, Mendelian randomization, and animal studies, case reports, conference abstracts, reviews or meta-analyses, and studies that did not distinguish AGA from other forms of alopecia or focused primarily on polycystic ovary syndrome-related hair loss. For the primary MetS analysis, the original report was also required to provide an identifiable diagnostic definition. For duplicate publications or overlapping cohorts, the more complete or more recent report was retained.

## 5 Data extraction

We extracted the first author, publication year, country or region, study design, sampling source, AGA diagnostic and grading criteria, MetS definition, age and sex distribution, and the number of cases and participants for MetS and its components. Components included abdominal obesity, hypertension or elevated blood pressure, diabetes mellitus, dyslipidemia, hypertriglyceridemia, elevated LDL-C, and low HDL-C. The triglyceride-to-HDL-C ratio and atherogenic index of plasma (AIP) were also examined when reported. AGA severity was categorized as mild, moderate, or severe according to the original study. Studies without reliably extractable severity data were excluded from that subgroup analysis.

## 6 Statistical analysis

Single-proportion meta-analyses were performed after logit transformation. Random-effects models were specified *a priori*, with between-study variance estimated using restricted maximum likelihood (REML); confidence intervals for individual studies were calculated using the Wilson method. Pooled prevalence estimates with 95% CIs were reported together with Cochran’s Q, *I*², and τ². Subgroup analyses by region, sex, AGA severity, and MetS definition were used to explore heterogeneity. Because some sex- and severity-specific strata were derived from the same study and regional subgroups often contained only one or two studies, these comparisons were considered descriptive. Sensitivity analyses used a leave-one-out approach; for MetS, additional analyses excluded the matched-sampling study and applied alternative estimators of τ². Egger’s test was reported only when an outcome included at least 10 studies. A two-sided P < 0.05 was used as a reference threshold for statistical significance. To assess the primary MetS outcome, an exploratory subgroup analysis was stratified by AHRQ quality. Sensitivity analysis then excluded low-quality studies.

## 7 Quality assessment

Cross-sectional studies and cross-sectional analyses of cohort baseline data were assessed using the 11-item Agency for Healthcare Research and Quality (AHRQ) checklist; the two retrospective patient cohorts were assessed using the Newcastle-Ottawa Scale (NOS). For the AHRQ checklist, each clearly satisfied item received 1 point, whereas ‘no,’ ‘unclear,’ or ‘not applicable’ received 0 points. Total scores of 8–11, 4–7, and 0–3 indicated high, moderate, and low quality, respectively. The NOS evaluated selection (up to 4 points), comparability (up to 2 points), and outcome assessment (up to 3 points); scores of 7–9, 4–6, and 0–3 indicated high, moderate, and low quality, respectively. Two reviewers assessed each study independently, and disagreements were resolved through discussion with a third reviewer. Because the two instruments differ in structure, their raw scores were not directly compared.

## 8 Results

### 8.1 Study selection and characteristics

Database searches yielded 2,241 records, and one additional record was identified through other sources, for a total of 2,242. After 2,068 records were excluded during title and abstract screening, 174 records underwent further assessment. A further 129 records were excluded because the study objective or design was ineligible, leaving 45 articles for full-text review. Seventeen full-text articles were excluded: 10 did not provide extractable data, five had ineligible objectives, one was suspected to be a duplicate publication, and one involved an overlapping target cohort. The systematic review ultimately included 28 studies (**Figure 1**). The number of studies and participants varied by outcome: MetS, 12 studies/3,623 participants; abdominal obesity, 8/3,718; hypertension or elevated blood pressure, 18/7,450; diabetes mellitus, 11/6,398; dyslipidemia, 12/6,582; hypertriglyceridemia, 7/1,451; elevated LDL-C, 2/73; and low HDL-C, 7/1,451. Study characteristics are summarized in **Table 1**.

**Figure 1:**
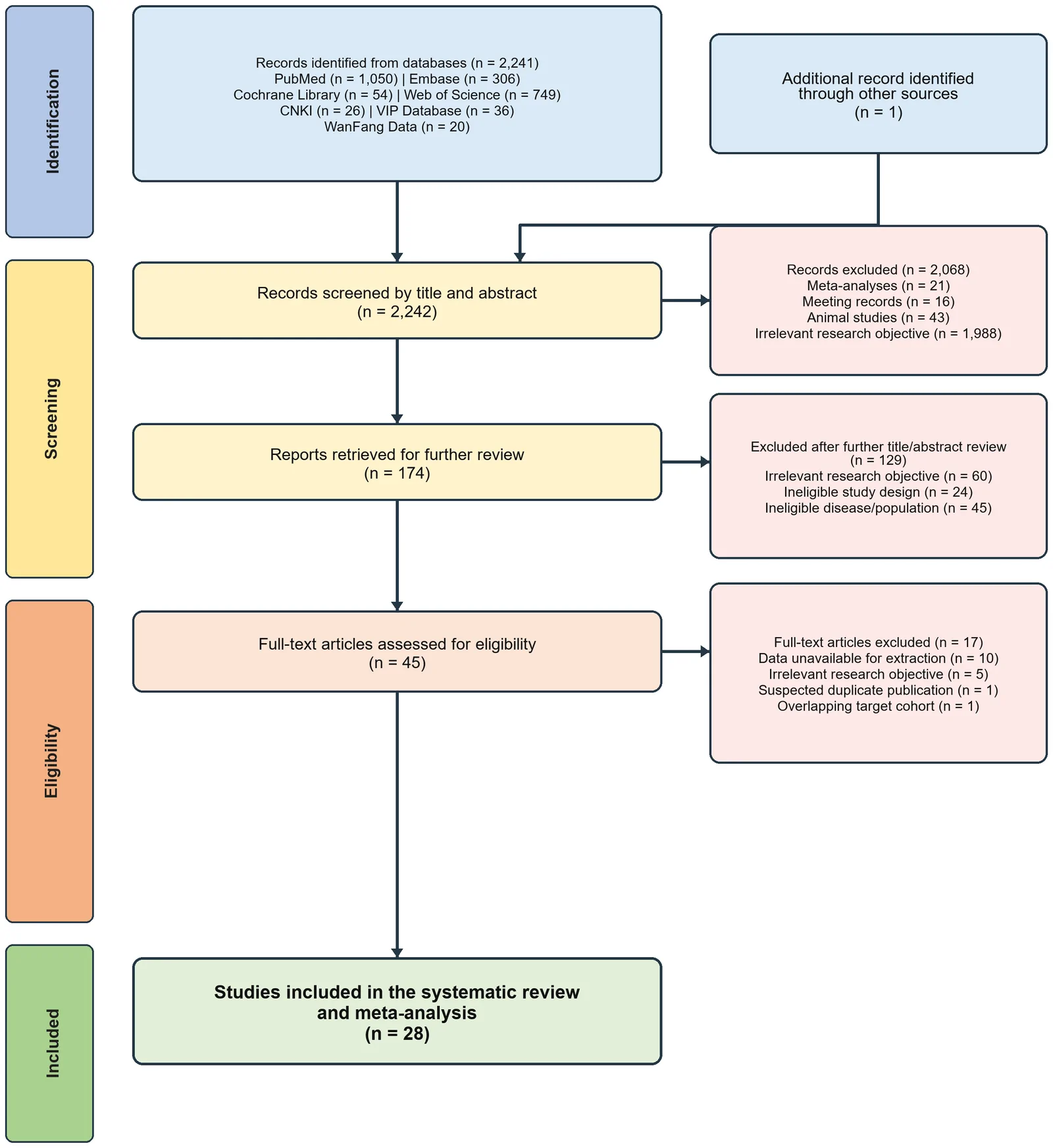
PRISMA flowchart of the review search.

**Table 1:** Characteristics of studies included.

Study	Country/ region	Study design	AGA definition	MetS definition	Sample characteristics	Overall MetS	MetS by sex	MetS by AGA severity	MetS components	Components by sex	Components by AGA severity
Agamia et al., 2016 (22)	Egypt (North Africa)	Hospital-based cross-sectional	Clinical diagnosis; Hamilton–Norwood classification	NCEP ATP III	n=300; age, years: 44.16 ± 8.48; male/female: 300/0	153/300 (51.0%)	male: 153/300 (51.0%)	Mild: 42/120 (35.0%); moderate: 45/84 (53.6%); severe: 66/96 (68.8%)	HTN/elevated BP: 51/300 (17.0%)	Male-only cohort; overall component values apply	NR
Fortes et al., 2017 (23)	Italy (Europe)	Hospital-based cross-sectional	Clinical diagnosis; Hamilton–Norwood (male) and Ludwig (female)	Not assessed	n=351; age, years: 35.6 ± 14.2; male/female: 237/114	NR	NR	NR	HTN/elevated BP: 28/350 (8.0%) Dyslipidemia: 17/350 (4.9%)	NR	Dyslipidemia: mild: 7/236 (3.0%); moderate/severe: 10/114 (8.8%)
Gopinath et al., 2016 (24)	India (South Asia)	Analytical cross-sectional	Early-onset AGA (<35 years); Norwood grade ≥III	IDF	n=85; age, years: 18–55; male/female: 85/0	19/85 (22.4%)	male: 19/85 (22.4%)	Norwood 3/3A/3V: 8/32 (25.0%); 4/4A: 2/22 (9.1%); grade ≥5: 9/31 (29.0%)	Central obesity: 32/85 (37.6%) HTN/elevated BP: 42/85 (49.4%) HTG: 25/85 (29.4%) Low HDL-C: 22/85 (25.9%)	Male-only cohort; overall component values apply	NR
Oiwoh et al., 2023 (25)	Nigeria (Africa)	Community-based cross-sectional	Clinical diagnosis; Hamilton–Norwood/Ludwig classification	IDF	n=260; age, years: 51.32 ± 16.31; male/female: NR	21/260 (8.1%)	NR	NR	Central obesity: 66/260 (25.4%) HTN/elevated BP: 48/260 (18.5%) DM: 14/260 (5.4%) Dyslipidemia: 160/260 (61.5%) HTG: 33/260 (12.7%) Low HDL-C: 130/260 (50.0%)	Central obesity: male, 41 events; female, 25 events (sex-specific denominators NR)	NR
Park et al., 2017 (15)	Republic of Korea (East Asia)	Health-screening cross-sectional	BASP classification	Korean/Asian-specific criteria	n=991; age, years: male: 59.1 ± 12.3; female: 61.1 ± 10.2; male/female: 613/378	259/991 (26.1%)	male: 179/613 (29.2%); female: 80/378 (21.2%)	Mild: 92/443 (20.8%); moderate: 108/381 (28.3%); severe: 59/167 (35.3%)	HTN/elevated BP: 346/991 (34.9%) DM: 182/991 (18.4%) Dyslipidemia: 92/991 (9.3%)	NR	HTN: mild/moderate/severe: 126/443, 144/381, 76/167 DM: 66/443, 82/381, 34/167 Dyslipidemia: 39/443, 38/381, 15/167
Sakpuwadol et al., 2023 (7)	Thailand (Southeast Asia)	Retrospective patient cohort	Clinical diagnosis: Ludwig, Olsen, or Hamilton–Norwood subtype	Not assessed	n=519; age, years: NR; male/female: 0/519	NR	NR	NR	HTN/elevated BP: 42/519 (8.1%) DM: 50/519 (9.6%) Dyslipidemia: 49/519 (9.4%)	Female-only cohort; overall component values apply	NR
Verma et al., 2024 (16)	India (South Asia)	Hospital-based cross-sectional	Clinical and trichoscopic diagnosis; Ludwig classification	NCEP ATP III	n=50; age, years: 18–45; male/female: 0/50	12/50 (24.0%)	female: 12/50 (24.0%)	Ludwig I: 1/30 (3.3%); II: 9/18 (50.0%); III: 2/2 (100%)	Central obesity: 15/50 (30.0%) HTN/elevated BP: 29/50 (58.0%) DM: 8/50 (16.0%) Dyslipidemia: 11/50 (22.0%) HTG: 9/50 (18.0%) Low HDL-C: 17/50 (34.0%) Elevated LDL-C: 31/50 (62.0%)	Female-only cohort; overall component values apply	NR
Asgher et al., 2024 (26)	Pakistan (South Asia)	Hospital-based cross-sectional	Clinical diagnosis; grading as reported	NCEP ATP III/study definition	n=95; age, years: 34.07 ± 6.36 (range 20–50); male/female: 95/0	16/95 (16.8%)	male: 16/95 (16.8%)	NR	Central obesity: 44/95 (46.3%) HTN/elevated BP: 29/95 (30.5%) HTG: 30/95 (31.6%) Low HDL-C: 15/95 (15.8%)	Male-only cohort; overall component values apply	NR
Devi et al., 2023 (27)	Pakistan (South Asia)	Hospital-based cross-sectional	Clinical diagnosis of male AGA	Not assessed	n=176; age, years: 46.9 ± 7.9; male/female: 176/0	NR	NR	NR	HTN/elevated BP: 103/176 (58.5%) DM: 45/176 (25.6%) Dyslipidemia: 114/176 (64.8%)	Male-only cohort; overall component values apply	NR
Hirsso et al., 2006 (28)	Finland (Northern Europe)	Population cohort, cross-sectional analysis	Extensive AGA: Hamilton–Norwood III vertex–VII	Not assessed	n=129; age, years: 63; male/female: 129/0	NR	NR	NR	HTN/elevated BP: 77/129 (59.7%) DM: 27/129 (20.9%) Dyslipidemia: 45/129 (34.9%)	Male-only cohort; overall component values apply	NR
Su et al., 2010 (29)	Taiwan, China (East Asia)	Community-based cross-sectional	Moderate-to-severe AGA: Norwood IV–VII	Study-specific criteria	n=150; age, years: 40–91; male/female: 150/0	32/150 (21.3%)	male: 32/150 (21.3%)	Norwood IV–VII: 32/150 (21.3%); V–VII: 21/93 (22.6%)	NR	NR	NR
Su et al., 2011 (30)	Taiwan, China (East Asia)	Occupational cross-sectional	AGA: Norwood grade ≥III	Not assessed	n=187; age, years: 38.6 ± 7.6 (range 20–64); male/female: 187/0	NR	NR	NR	NR	NR	NR
Yi et al., 2012 (31)	Republic of Korea (East Asia)	Population cohort, cross-sectional analysis	male: Norwood ≥IV; female: Ludwig ≥I	Study-specific criteria	n=553; age: middle-aged population; male/female: 332/221	209/553 (37.8%)	male: 102/332 (30.7%); female: 107/221 (48.4%)	NR	NR	NR	NR
Majella et al., 2025 (8)	India (South Asia)	Descriptive cross-sectional	Early-onset AGA (onset ≤30 years); Hamilton–Norwood	NCEP ATP III	n=100; age, years: 18–40; male/female: 100/0	63/100 (63.0%)	male: 63/100 (63.0%)	Mild/moderate: 38/70 (54.3%); severe: 25/30 (83.3%)	Central obesity: 75/100 (75.0%) HTN/elevated BP: 76/100 (76.0%) HTG: 41/100 (41.0%) Low HDL-C: 65/100 (65.0%)	Male-only cohort; overall component values apply	Central obesity: mild/moderate: 47/70; severe: 28/30 Elevated BP: 50/70; 26/30 HTG: 23/70; 18/30 Low HDL-C: 44/70; 21/30
Zhu et al., 2022 (18)	China (East Asia)	Community-based cross-sectional	Hamilton–Norwood (male); Ludwig (female)	Chinese Diabetes Society/study definition	n=762; age, years: 54.95 ± 8.43 (range 22–85); male/female: 646/116	225/762 (29.5%)	male: 193/646 (29.9%); female: 32/116 (27.6%)	male, three reported severity strata: 80/269, 60/217, 53/160; female: 8/39, 18/61, 6/16	Central obesity: 306/762 (40.2%) HTN/elevated BP: 395/762 (51.8%) DM: 132/762 (17.3%) Dyslipidemia: 178/762 (23.4%) HTG: 137/762 (18.0%) Low HDL-C: 51/762 (6.7%)	NR	NR
Legiawati et al., 2024 (32)	Indonesia (Southeast Asia)	Hospital-based cross-sectional	Hamilton–Norwood (male); Ludwig (female); early versus usual onset	Not assessed	n=40; age, years: NR; male/female: NR	NR	NR	NR	HTN/elevated BP: 6/40 (15.0%) DM: 3/40 (7.5%) Dyslipidemia: 5/40 (12.5%)	NR	NR
Husseinali et al., 2026 (33)	United States (North America)	National cohort database, matched cross-sectional analysis	AGA diagnosis concepts/codes in electronic health records	Not assessed	n=1,015; age, years: 60.62 ± 16.76; male/female/unknown: 296/694/25	NR	NR	NR	HTN/elevated BP: 517/1015 (50.9%) DM: 236/1015 (23.3%) Dyslipidemia: 615/1015 (60.6%)	NR	NR
Özcan et al., 2022 (12)	Türkiye (West Asia)	Retrospective patient cohort	Clinical pattern plus trichoscopy (>20% hair-shaft diameter diversity)	Pediatric/adolescent cardiometabolic risk criteria	n=23; age, years: 15.3 ± 2.1 (range 10–21); male/female: 11/12	NR	NR	NR	HTN/elevated BP: 1/23 (4.3%) Elevated LDL-C: 2/23 (8.7%)	NR	NR
Si et al., 2020 (34)	China (East Asia)	School-based cross-sectional	Clinical diagnosis; Hamilton–Norwood (male) and Ludwig (female)	Not assessed	n=109; age, years: 18–30; male/female: 64/45	NR	NR	NR	NR	NR	NR
Trevisan et al., 1993 (35)	Italy (Europe)	Cross-sectional analysis of longitudinal cohort examinations	Frontal or fronto-occipital male-pattern baldness	Not assessed	n=553; age, years: NR; male/female: 553/0	NR	NR	NR	HTN/elevated BP: 29.9% (n NR) Dyslipidemia: 165/553 (29.8%)*	Male-only cohort; overall component values apply	NR
Hirsso et al., 2005 (36)	Finland (Northern Europe)	Population cohort, cross-sectional analysis	AGA: Ludwig II–III	Not assessed	n=105; age, years: 63; male/female: 0/105	NR	NR	NR	DM: 14% (n NR)	female-only cohort; overall component values apply	NR
Yang et al., 2014 (37)	Taiwan, China (East Asia)	Retrospective cross-sectional	Clinical/photographic diagnosis; Hamilton–Norwood or female-pattern type in male participants	Not assessed	n=189; age, years: 30.8 ± 10.2 (range 18–64); male/female: 189/0	NR	NR	NR	HTN/elevated BP: 14/189 (7.4%) DM: 4/189 (2.1%)	Male-only cohort; overall component values apply	NR
Salman et al., 2017 (38)	Türkiye (West Asia)	Hospital-based cross-sectional	Hamilton–Norwood (male); Ludwig (female)	Not assessed	n=409; age, years: NR; male/female: 281/128	NR	NR	NR	NR	NR	NR
Bazmi et al., 2025 (39)	Iran (West Asia)	Cohort baseline cross-sectional analysis	Researcher-assessed AGA	Not assessed; study evaluated MASLD	n=6,004; age, years: 49.47 ± 9.63 (range 35–70); male/female: 2697/3307	NR	NR	NR	NR	NR	NR
Girishma et al., 2025 (40)	India (South Asia)	Hospital-based cross-sectional	Early-onset male AGA (18–35 years); grading as reported	NCEP ATP III	n=99; age, years: 24.84 ± 4.85 (range 18–35); male/female: 99/0	7/99 (7.1%)	male: 7/99 (7.1%)	Mild: 7/94 (7.4%); severe: 0/5 (0%)	Central obesity: 9/99 (9.1%) HTN/elevated BP: 34/99 (34.3%) HTG: 22/99 (22.2%) Low HDL-C: 28/99 (28.3%)	Male-only cohort; overall component values apply	Elevated BP: mild: approximately 32/94; severe: 2/5
Memon et al., 2021 (41)	Pakistan (South Asia)	Hospital-based cross-sectional	Hamilton–Norwood classification	NCEP ATP III/AHA-related criteria	n=178; age, years: 39.08 ± 10.14 (range 18–60); male/female: 178/0	18/178 (10.1%)	male: 18/178 (10.1%)	NR	NR	NR	NR
Goswami et al., 2024 (42)	India (South Asia)	Descriptive cross-sectional	Clinical male AGA; Hamilton–Norwood classification	Not assessed	n=100; age, years: 27.12 ± 5.78; male/female: 100/0	NR	NR	NR	NR	NR	NR
He et al., 2026 (43)	China (East Asia)	Large population-based cross-sectional	Dermatologist-diagnosed AGA; BASP classification	Not assessed	n=2,267; age, years: ≥18; male/female: 1813/454	NR	NR	NR	Central obesity: 829/2267 (36.6%) HTN/elevated BP: 759/2267 (33.5%) DM: 316/2267 (13.9%) Dyslipidemia: 950/2267 (41.9%)	NR	NR

AGA, androgenetic alopecia; AHA, American Heart Association; BASP, Basic and Specific classification; BMI, body mass index; BP, blood pressure; DM, diabetes mellitus; FPHL, female pattern hair loss; HDL-C, high-density lipoprotein cholesterol; HTG, hypertriglyceridemia; HTN, hypertension; IDF, International Diabetes Federation; LDL-C, low-density lipoprotein cholesterol; MASLD, metabolic dysfunction-associated steatotic liver disease; MetS, metabolic syndrome; NCEP ATP III, National Cholesterol Education Program Adult Treatment Panel III; NR, not reported.

### 8.2 Study quality

Among the 28 included studies, 26 cross-sectional studies or cross-sectional analyses of cohort baseline data were assessed using the AHRQ checklist: 11 were rated as high quality, 14 as moderate quality, and one as low quality. Two retrospective patient cohorts were assessed using the NOS: one was rated as moderate quality and one as low quality. When the category assigned by the relevant instrument was summarized, 11 studies were rated as high quality, 15 as moderate quality, and two as low quality (**Table 2**). Among the 12 studies in our primary MetS analysis, four high-quality studies (725 cases/2,456 participants) yielded a pooled prevalence of 28.8% (95% CI, 22.8%–35.7%; I² = 89.3%; τ² = 0.090). Seven moderate-quality studies (297/1,117) yielded a pooled prevalence of 20.9% (95% CI, 9.6%–39.6%; I² = 97.1%; τ² = 1.439). A single low-quality study reported a prevalence of 24.0% (12/50; 95% CI, 14.3%–37.4%). The test for differences between quality strata did not indicate significance (Q = 3.01, df = 2, P = 0.222), and the comparison was considered exploratory (**Supplementary Figure 1**).

**Table 2:** Quality assessment of the studies included in the systematic review and meta-analysis.

Study	Study design	Assessment tool	Selection (NOS)	Comparability (NOS)	Outcome (NOS)	Total score	Quality rating
Agamia (2016) (22)	Hospital-based cross-sectional study	AHRQ	—	—	—	4	Moderate
Fortes (2017) (23)	Hospital-based cross-sectional study	AHRQ	—	—	—	7	Moderate
Gopinath (2016) (24)	Analytical cross-sectional study	AHRQ	—	—	—	6	Moderate
Oiwoh (2023) (25)	Community-based cross-sectional study	AHRQ	—	—	—	5	Moderate
Park (2017) (15)	Health-examination cross-sectional study	AHRQ	—	—	—	8	High
Sakpuwadol (2023) (7)	Retrospective patient cohort	NOS	2	1	2	5	Moderate
Verma (2024) (16)	Hospital-based cross-sectional study	AHRQ	—	—	—	3	Low
Asgher (2024) (26)	Hospital-based cross-sectional study	AHRQ	—	—	—	6	Moderate
Devi (2023) (27)	Hospital-based cross-sectional study	AHRQ	—	—	—	7	Moderate
Hirsso (2006) (28)	Cross-sectional analysis of a population cohort	AHRQ	—	—	—	8	High
Su (2010) (29)	Community-based cross-sectional study	AHRQ	—	—	—	9	High
Su (2011) (30)	Occupational cross-sectional study	AHRQ	—	—	—	9	High
Yi (2012) (31)	Cross-sectional analysis of a population cohort	AHRQ	—	—	—	8	High
Majella (2025) (8)	Descriptive cross-sectional study	AHRQ	—	—	—	4	Moderate
Zhu (2022) (20)	Community-based cross-sectional study	AHRQ	—	—	—	8	High
Legiawati (2024) (32)	Hospital-based cross-sectional study	AHRQ	—	—	—	6	Moderate
Husseinali (2026) (33)	Matched cross-sectional database analysis	AHRQ	—	—	—	8	High
Özcan (2022) (12)	Retrospective patient cohort	NOS	2	0	1	3	Low
Si (2020) (34)	School-based cross-sectional survey	AHRQ	—	—	—	9	High
Trevisan (1993) (35)	Cross-sectional analysis of longitudinal examinations	AHRQ	—	—	—	6	Moderate
Hirsso (2005) (36)	Cross-sectional analysis of a population cohort	AHRQ	—	—	—	8	High
Yang (2014) (37)	Retrospective cross-sectional study	AHRQ	—	—	—	7	Moderate
Salman (2017) (38)	Hospital-based cross-sectional study	AHRQ	—	—	—	6	Moderate
Bazmi (2025) (39)	Cross-sectional analysis of cohort baseline data	AHRQ	—	—	—	9	High
Girishma (2025) (40)	Hospital-based cross-sectional study	AHRQ	—	—	—	5	Moderate
Memon (2021) (41)	Hospital-based cross-sectional study	AHRQ	—	—	—	4	Moderate
Goswami (2024) (42)	Descriptive cross-sectional study	AHRQ	—	—	—	4	Moderate
He (2026) (43)	Large population-based cross-sectional study	AHRQ	—	—	—	9	High

AHRQ, Agency for Healthcare Research and Quality; NOS, Newcastle–Ottawa Scale.

### 8.3 Pooled prevalence and subgroup analyses

Twelve studies included 3,623 patients with AGA, of which 1,034 met the diagnostic criteria for MetS. The pooled prevalence under the random-effects model was 23.7% (95% CI, 15.5%–34.5%), with substantial heterogeneity (I² = 95.4%; τ² = 0.816) and a 95% PI of 3.7%–71.7% (**Figure 2**; **Table 3**). In the revised exploratory analysis by MetS definition (**Figure 3**), the pooled prevalence was 24.4% for NCEP ATP III-based criteria (6 studies; 822 participants; 95% CI, 10.7%–46.4%), 13.6% for IDF criteria (2 studies; 345 participants; 95% CI, 4.7%–33.6%), and 28.8% for ethnicity- or study-specific criteria (4 studies; 2,456 participants; 95% CI, 22.8%–35.7%).

**Figure 2:**
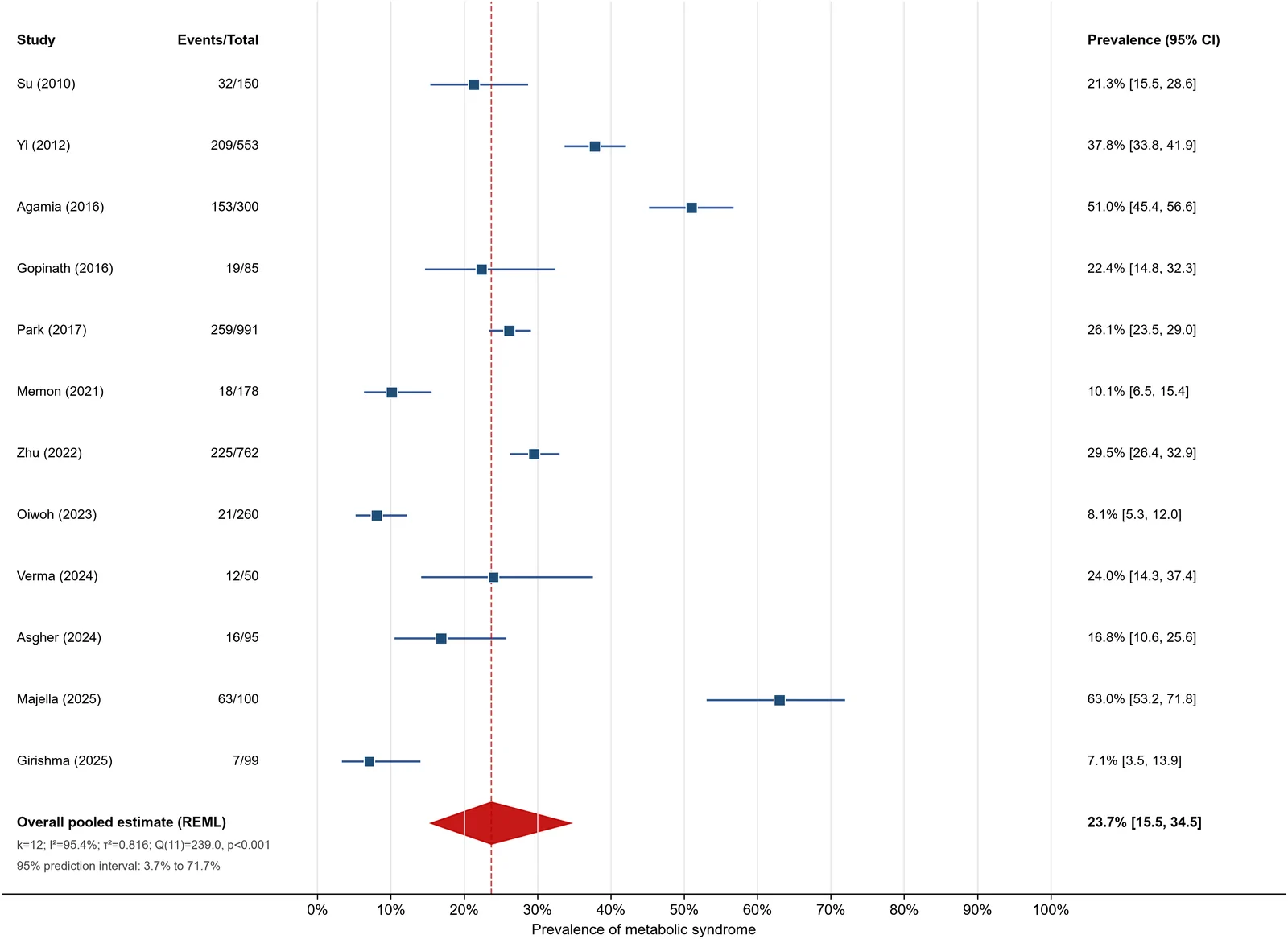
Forest plot of the prevalence of MetS in AGA patients.

**Table 3:** Pooled prevalence of MetS and its components among patients with AGA.

Outcome	No. of studies	Participants	Cases	Pooled prevalence, % (95% CI)	I², %	τ²	P for heterogeneity	95% PI, %	Egger’s test, P
Metabolic syndrome	12	3,623	1,034	23.7 (15.5-34.5)	95.4	0.816	<0.001	3.7-71.7	0.242
Abdominal obesity	8	3,718	1,376	36.0 (23.0-51.5)	92.9	0.786	<0.001	5.3-85.0	Not performed
Hypertension	18	7,450	2,597	30.7 (20.6-43.1)	97.5	1.267	<0.001	3.7-83.8	0.353
Diabetes mellitus	11	6,398	1,017	13.3 (9.0-19.2)	91.5	0.478	<0.001	2.9-44.2	0.284
Dyslipidemia	12	6,582	2,237	24.5 (13.5-40.3)	98.8	1.583	<0.001	1.7-85.8	0.210
Hypertriglyceridemia	7	1,451	297	23.6 (17.0-31.7)	87.3	0.253	<0.001	7.1-55.6	Not performed
Elevated LDL-C	2	73	33	30.0 (2.6-87.3)	92.2	3.719	<0.001	NA	Not performed
Low HDL-C	7	1,451	328	28.8 (15.4-47.4)	97.7	1.118	<0.001	2.1-88.2	Not performed

Egger’s test was reported only when at least 10 studies were available. For outcomes with fewer than three studies, a 95% PI was not estimated.

AGA, androgenetic alopecia; CI, confidence interval; HDL-C, high-density lipoprotein cholesterol; LDL-C, low-density lipoprotein cholesterol; MetS, metabolic syndrome; NA, not applicable/not estimable; PI, prediction interval; REML, restricted maximum likelihood; τ², between-study variance.

**Figure 3:**
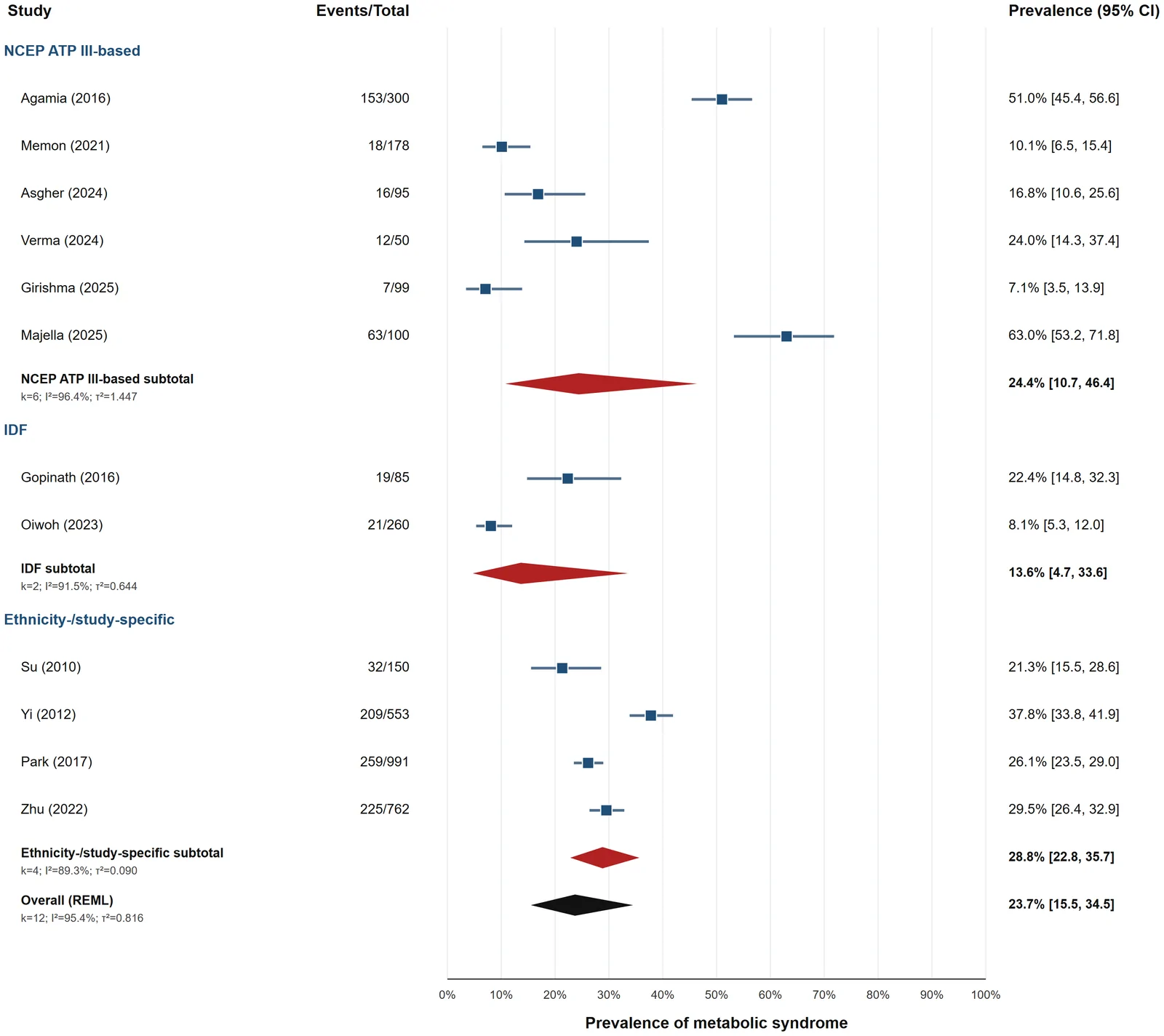
Subgroup analysis based on MetS diagnostic criteria.

In regional subgroup analyses of MetS (**Table 4**; **Supplementary Figure 2**), pooled prevalence was 23.4% in Africa (2 studies; 560 participants), 28.8% in East Asia (4 studies; 2,456 participants), and 20.6% in South Asia (6 studies; 607 participants); the CI for Africa was wide (2.6%–77.5%). The corresponding estimates were 25.8% for men (10 studies; 2,598 participants) and 29.8% for women (4 studies; 765 participants) (**Table 4**; **Supplementary Figure 3**). By AGA severity, pooled prevalence was 19.7% for mild AGA (6 studies; 1,027 participants), 30.8% for moderate AGA (6 studies; 840 participants), and 44.2% for severe AGA (8 studies; 600 participants) (**Table 4**; **Supplementary Figure 4**). The female stratum was supported by only four studies (765 participants), compared with 10 studies (2,598 participants) supporting the male stratum. The small numerical difference was thus not interpreted as evidence of a sex effect.

**Table 4:** Subgroup analyses of the prevalence of MetS and its components among patients with AGA.

Outcome/ subgroup factor	Subgroup	No. of studies	Participants	Cases	Pooled prevalence, % (95% CI)	I², %	τ²	P for heterogeneity
Metabolic syndrome Region	Africa	2	560	174	23.4 (2.6-77.5)	98.9	3.023	<0.001
	East Asia	4	2,456	725	28.8 (22.8-35.7)	89.3	0.090	<0.001
	South Asia	6	607	135	20.6 (9.7-38.3)	94.9	1.111	<0.001
Metabolic syndrome Sex	Male	10	2,598	782	25.8 (16.5-37.8)	94.5	0.775	<0.001
	Female	4	765	231	29.8 (19.2-43.1)	93.8	0.308	<0.001
Metabolic syndrome AGA severity	Mild	6	1,027	238	19.7 (11.4-31.8)	83.6	0.508	<0.001
	Moderate	6	840	253	30.8 (19.6-44.8)	84.1	0.450	<0.001
	Severe	8	600	241	44.2 (26.1-64.0)	89.6	1.048	<0.001
Metabolic syndrome Study quality	High	4	2,456	725	28.8 (22.8-35.7)	89.3	0.090	<0.001
	Moderate	7	1,117	297	20.9 (9.6-39.6)	97.1	1.439	<0.001
	Low	1	50	12	24.0 (14.3-37.4)	NA	NA	NA
Abdominal obesity Region	Africa	1	260	66	25.4 (20.5-31.0)	NA	NA	NA
	East Asia	2	3,029	1,135	38.1 (34.7-41.6)	68.1	0.008	0.077
	South Asia	5	429	175	37.2 (17.0-63.3)	94.6	1.405	<0.001
Abdominal obesity Sex	Male	4	379	160	39.0 (14.1-71.3)	95.7	1.857	<0.001
	Female	1	50	15	30.0 (19.0-44.0)	NA	NA	NA
Hypertension Region	Africa	2	560	99	17.7 (14.7-21.1)	0.0	0.000	0.651
	Europe	2	479	105	26.4 (2.2-85.3)	99.1	3.983	<0.001
	East Asia	4	4,209	1,514	28.6 (12.2-53.6)	97.8	1.149	<0.001
	Southeast Asia	2	559	48	9.9 (5.5-17.3)	54.1	0.131	0.140
	South Asia	6	605	313	51.3 (37.1-65.3)	90.6	0.474	<0.001
	West Asia	1	23	1	4.3 (0.6-25.2)	NA	NA	NA
	North America	1	1,015	517	50.9 (47.9-54.0)	NA	NA	NA
Hypertension Sex	Male	8	1,173	426	38.7 (21.8-58.8)	96.7	1.341	<0.001
	Female	2	569	71	25.7 (2.3-83.7)	98.6	3.734	<0.001
Hypertension AGA severity	Mild/moderate	2	894	320	52.0 (17.9-84.3)	97.2	1.299	<0.001
	Severe	2	197	102	68.6 (22.7-94.2)	92.6	1.949	<0.001
Diabetes mellitus Region	Africa	1	260	14	5.4 (3.2-8.9)	NA	NA	NA
	Europe	1	129	27	20.9 (14.8-28.8)	NA	NA	NA
	East Asia	4	4,209	634	11.1 (4.5-24.6)	90.1	0.905	<0.001
	Southeast Asia	2	559	53	9.5 (7.3-12.2)	0.0	0.000	0.658
	South Asia	2	226	53	22.1 (14.1-32.8)	48.6	0.085	0.163
	North America	1	1,015	236	23.3 (20.8-26.0)	NA	NA	NA
Diabetes mellitus Sex	Male	3	494	76	11.7 (2.4-41.4)	92.5	2.076	<0.001
	Female	2	569	58	11.3 (7.0-17.8)	49.2	0.083	0.160
Dyslipidemia Region	Africa	1	260	160	61.5 (55.5-67.3)	NA	NA	NA
Dyslipidemia Region	Europe	2	479	62	14.3 (1.6-62.5)	98.3	2.715	<0.001
	East Asia	3	4,020	1,220	22.1 (8.6-46.2)	99.4	0.950	<0.001
	Southeast Asia	2	559	54	9.7 (7.5-12.4)	0.0	0.000	0.530
	South Asia	2	226	125	42.5 (10.5-82.2)	96.0	1.687	<0.001
	West Asia	1	23	1	4.3 (0.6-25.2)	NA	NA	NA
	North America	1	1,015	615	60.6 (57.5-63.6)	NA	NA	NA
Dyslipidemia Sex	Male	2	305	159	49.9 (22.9-76.9)	96.1	0.731	<0.001
	Female	2	569	60	14.1 (5.8-30.2)	86.0	0.426	0.008
Hypertriglyceridemia Region	Africa	1	260	33	12.7 (9.2-17.3)	NA	NA	NA
	East Asia	1	762	137	18.0 (15.4-20.9)	NA	NA	NA
	South Asia	5	429	127	28.8 (21.8-37.0)	66.3	0.117	0.018
Hypertriglyceridemia Sex	Male	4	379	118	31.0 (23.8-39.2)	63.3	0.086	0.043
	Female	1	50	9	18.0 (9.6-31.1)	NA	NA	NA
Elevated LDL-C Region	South Asia	1	50	31	62.0 (48.0-74.3)	NA	NA	NA
	West Asia	1	23	2	8.7 (2.2-28.9)	NA	NA	NA
Elevated LDL-C Sex	Female	1	50	31	62.0 (48.0-74.3)	NA	NA	NA
Low HDL-C Region	Africa	1	260	130	50.0 (44.0-56.0)	NA	NA	NA
	East Asia	1	762	51	6.7 (5.1-8.7)	NA	NA	NA
	South Asia	5	429	147	32.5 (18.5-50.5)	92.6	0.669	<0.001
Low HDL-C Sex	Male	4	379	130	32.1 (15.3-55.2)	94.4	0.900	<0.001
	Female	1	50	17	34.0 (22.3-48.0)	NA	NA	NA

Random-effects meta-analysis of logit-transformed proportions using REML. For single-study strata, I², τ², and P for heterogeneity were not estimable and are shown as NA. Egger’s tests were not repeated within subgroups because most strata included fewer than 10 studies. Study-quality strata were defined using AHRQ ratings for the 12 studies contributing to the primary MetS analysis. AGA, androgenetic alopecia; CI, confidence interval; HDL-C, high-density lipoprotein cholesterol; LDL-C, low-density lipoprotein cholesterol; MetS, metabolic syndrome; NA, not applicable/not estimable; REML, restricted maximum likelihood; τ², between-study variance.

When stratified by country (**Figure 4**; **Supplementary Figure 5**), MetS prevalence was 25.9% in China (2 studies; 912 participants; 95% CI, 18.7%–34.8%), 51.0% in Egypt (1 study; 300 participants; 95% CI, 45.4%–56.6%), 25.3% in India (4 studies; 334 participants; 95% CI, 8.8%–54.1%), 8.1% in Nigeria (1 study; 260 participants; 95% CI, 5.3%–12.0%), 13.0% in Pakistan (2 studies; 273 participants; 95% CI, 7.7%–21.0%), and 31.6% in South Korea (2 studies; 1,544 participants; 95% CI, 21.4%–44.0%). Egypt and Nigeria were each represented by a single study, and heterogeneity was high in the Indian and South Korean subgroups; these country-level differences should therefore be interpreted as exploratory. The Chinese subgroup included studies conducted in Taiwan and Jiangsu. Because the two Chinese studies were limited to Jiangsu and Taiwan, their pooled estimates should not be interpreted as nationally representative of China.

**Figure 4:**
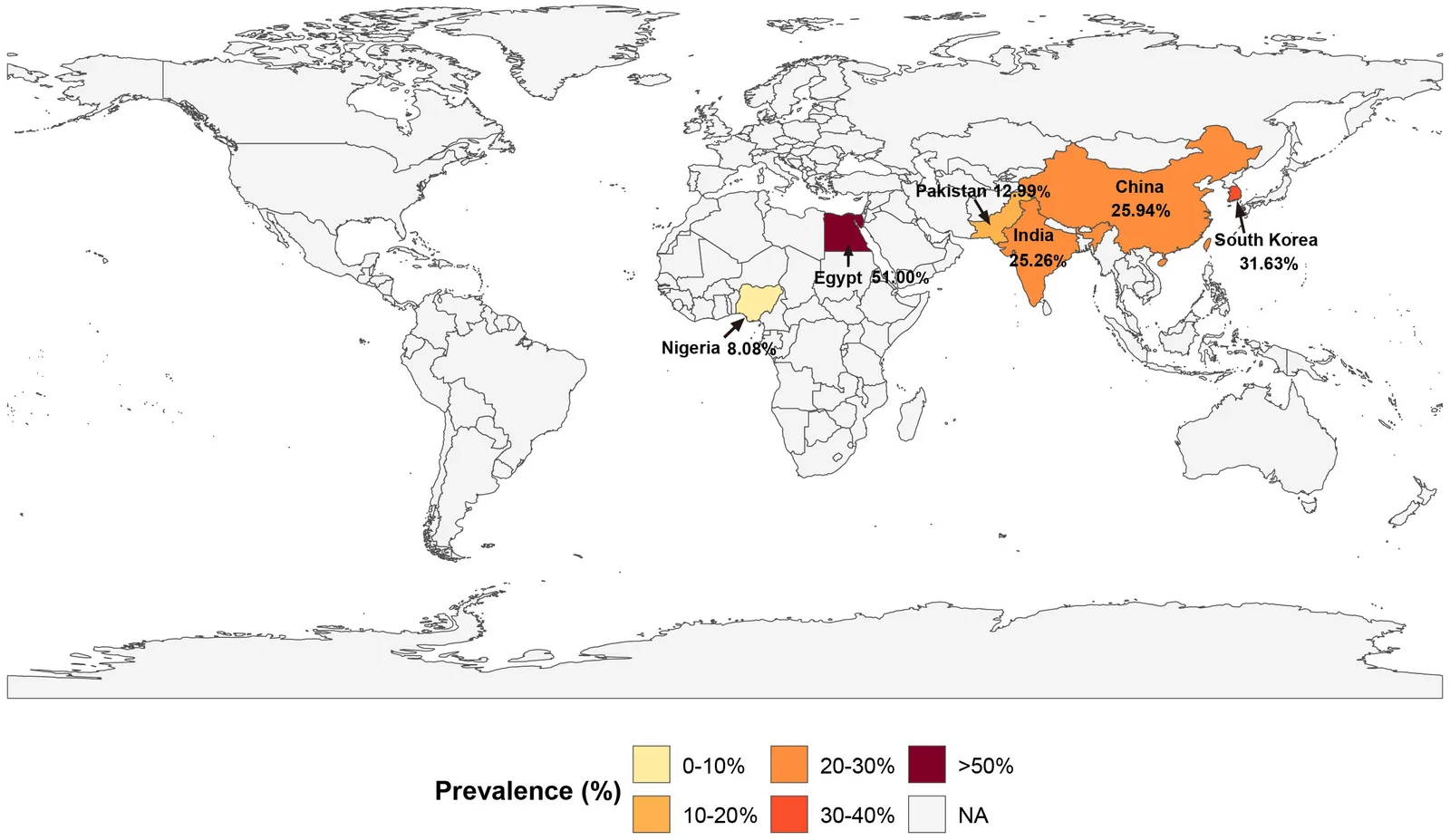
World map showing the prevalence rates of MetS by country.

The pooled prevalence of abdominal obesity was 36.0% (8 studies; 3,718 participants; 1,376 events; 95% CI, 23.0%–51.5%; I² = 92.9%; τ² = 0.786; 95% PI, 5.3%–85.0%) (**Supplementary Figure 6**). Regional estimates were 25.4% in Africa, 38.1% in East Asia, and 37.2% in South Asia; however, Africa was represented by one study, and heterogeneity was high in South Asia (**Supplementary Figure 7**). The pooled prevalence was 39.0% among men (4 studies); the estimate among women was 30.0% but was based on a single study (**Supplementary Figure 8**). Available data were insufficient for a reliable severity subgroup meta-analysis of abdominal obesity.

The pooled prevalence of hypertension or elevated blood pressure was 30.7% (18 studies; 7,450 participants; 2,597 events; 95% CI, 20.6%–43.1%; I² = 97.5%; τ² = 1.267; 95% PI, 3.7%–83.8%) (**Supplementary Figure 9**). Regional estimates varied widely, and subgroups for Europe, West Asia, and North America contained only one or two studies (**Supplementary Figure 10**). The pooled prevalence was 38.7% among men (8 studies) and 25.7% among women (2 studies), with substantial heterogeneity in both subgroups (**Supplementary Figure 11**). The severity analysis included only two studies and was therefore not used for inferential conclusions.

The pooled prevalence of diabetes mellitus was 13.3% (11 studies; 6,398 participants; 1,017 events; 95% CI, 9.0%–19.2%; I² = 91.5%; τ² = 0.478; 95% PI, 2.9%–44.2%) (**Supplementary Figure 12**). The corresponding estimates were 11.7% among men (3 studies) and 11.3% among women (2 studies); most regional subgroups contained few studies (**Supplementary Figure 12**).

The pooled prevalence of dyslipidemia was 24.5% (12 studies; 6,582 participants; 2,237 events; 95% CI, 13.5%–40.3%; I² = 98.8%; τ² = 1.583; 95% PI, 1.7%–85.8%) (**Supplementary Figure 13**). Definitions of dyslipidemia differed across studies, and estimates varied by region and sex. The pooled prevalence of hypertriglyceridemia was 23.6% (7 studies; 1,451 participants; 95% CI, 17.0%–31.7%; I² = 87.3%; τ² = 0.253; 95% PI, 7.1%–55.6%) (**Supplementary Figure 13**), and that of low HDL-C was 28.8% (7 studies; 1,451 participants; 95% CI, 15.4%–47.4%; I² = 97.7%; τ² = 1.118; 95% PI, 2.1%–88.2%) (**Supplementary Figure 13**). Only two studies, comprising 73 participants, reported elevated LDL-C; the pooled prevalence was 30.0% (95% CI, 2.6%–87.3%; I² = 92.2%; τ² = 3.719), and no further subgroup analysis was undertaken (**Supplementary Figure 13**). Most lipid subgroups by region or sex were informed by a single or few studies and were not used for definitive comparisons. No prevalence data suitable for pooling were available for the triglyceride-to-HDL-C ratio or AIP.

## 9 Sensitivity analysis

After each study was omitted in turn, pooled prevalence ranged from 21.1% to 25.9% for MetS, 31.2% to 41.3% for abdominal obesity, 28.4% to 33.0% for hypertension or elevated blood pressure, 12.3% to 15.3% for diabetes mellitus, 21.6% to 28.0% for dyslipidemia, 21.1% to 26.1% for hypertriglyceridemia, and 23.9% to 35.4% for low HDL-C. Thus, no single study fully determined the overall magnitude of these estimates, although substantial heterogeneity persisted. Because elevated LDL-C was reported in only two studies, omitting either study left a single estimate and did not permit a meaningful assessment of robustness. Excluding the single low-quality study yielded a pooled MetS prevalence of 23.6% (11 studies; 3,573 participants; 95% CI, 14.8%–35.5%; I² = 95.8%; τ² = 0.899), indicating little change in the point estimate but persistent heterogeneity.

## 10 Small-study effects and publication bias

Egger’s test was applied to outcomes with at least 10 studies. The P values were 0.242 for MetS, 0.353 for hypertension or elevated blood pressure, 0.284 for diabetes mellitus, and 0.210 for dyslipidemia (**Supplementary Figure 14**), providing no statistical evidence of small-study effects. This finding does not establish the absence of publication bias because Egger’s test has limited applicability in single-proportion meta-analysis and limited power when the number of studies is close to 10. Formal Egger testing and definitive interpretation of funnel-plot symmetry were not undertaken for abdominal obesity, hypertriglyceridemia, elevated LDL-C, or low HDL-C because fewer than 10 studies were available.

## 11 Discussion

AGA is mediated by genetic susceptibility and heightened androgen sensitivity of hair follicles, leading to the progressive conversion of terminal scalp hairs into vellus-like hairs. Although AGA is not life-threatening, it can markedly affect psychosocial well-being (44). Accumulating evidence has demonstrated that the pathogenesis of AGA involves multifactorial mechanisms. Abnormally elevated levels of intrafollicular 5-α-reductase and androgen receptors exert a decisive impact on the progression of AGA (45). Androgens drive follicular miniaturization by progressively shortening the anagen phase of hair growth, whereas estrogens may confer protective effects against this process (46). Recent investigations have further identified additional contributors to AGA, including perifollicular microinflammation, altered oxidative stress homeostasis, and depletion of hair follicle stem cells. Hair follicle development and regeneration are modulated by a panel of signaling cascades, such as the Wnt/β-catenin, BMP, Notch, and SIRT1/JNK/p38 MAPK pathways (47, 48). Under oxidative stress (OS) conditions, paraoxonase 1—an enzyme synthesized in the liver and bound to high-density lipoproteins to suppress lipid peroxidation—exhibits markedly reduced concentrations in patients with AGA. This observation provides supplementary evidence supporting the involvement of oxidative stress in AGA pathogenesis (49).

AGA may coexist with several conditions, including MetS, a cluster of risk factors comprising abdominal obesity, insulin resistance, hypertension, dyslipidemia (elevated triglycerides and reduced HDL-C), and elevated fasting glucose (50). Comprehensive examinations for male AGA patients, including atherosclerotic plaque detection, can reduce the risk of MetS-related complications (51). Obesity-induced inflammatory signaling in stem cells has been shown to suppress regenerative signaling and accelerate tissue miniaturization, underscoring the importance of preventing obesity-related organ dysfunction (52). AGA has also been associated with dyslipidemia, particularly in patients with more severe disease (53). Low HDL-C, uric acid, and 25-hydroxyvitamin D [25(OH)D] may be independent risk factors for early-onset MAGA (54), although the relevant studies did not provide clear prevalence data. MetS is a metabolic disorder whose prevalence is increasing worldwide in parallel with obesity; it traditionally encompasses obesity, hyperlipidemia, hyperinsulinemia, hypertension, and hyperglycemia (55, 56). Previous reports suggest that MetS is associated with skin diseases including psoriasis, atopic dermatitis, and acne vulgaris (57, 58).

This revised review includes 28 cross-sectional or cohort studies. The primary MetS analysis includes 12 studies and 3,623 patients with AGA, yielding a pooled prevalence of 23.7%. Among the individual components, pooled prevalence estimates were 36.0% for abdominal obesity, 30.7% for hypertension or elevated blood pressure, 28.8% for low HDL-C, and 24.5% for dyslipidemia. Previous population-based studies from the United States, Nigeria, and Tunisia reported MetS prevalence estimates of approximately 19.4%–25.9% (59–61); however, differences in age distribution, diagnostic criteria, and sampling regions preclude unadjusted direct comparisons with our findings.

Substantial between-study heterogeneity was observed in the prevalence of MetS among patients with AGA. Regional estimates ranged from 20.6% to 28.8%, although the African subgroup included only two studies and had a wide CI; estimates by sex were similar. MetS prevalence increased from 19.7% in mild AGA to 44.2% in severe AGA. Although clinically suggestive, this gradient may reflect differences in grading systems and confounding by age, obesity, and other factors. Estimates also varied across MetS definitions, but the ethnicity- or study-specific subgroup overlapped completely with the East Asian subgroup, making it difficult to distinguish the effect of diagnostic definition from that of region. The numerical difference between sexes should not be interpreted as a sex effect because the female stratum contained only four studies and 765 participants, compared with 10 studies and 2,598 participants in the male stratum. These data do not justify the use of female-specific screening protocols. The Chinese estimate was derived from regional studies in Jiangsu and Taiwan and cannot be generalized to the national population. Study quality strata also remained heterogeneous. All four high-quality studies were from East Asia, while the low-quality stratum contained only one study. Apparent quality-related differences may thus reflect confounding by geography and sampling setting.

Findings for MetS components suggest that patients presenting with AGA may also have metabolic abnormalities requiring routine clinical attention. In alopecia treatment, apart from medications such as finasteride and minoxidil solution, some studies have described certain complementary and integrative therapies, including clinical case-based experiences with Shenyang Sanhuo Decoction (62). For patients who are older, overweight, or abdominally obese, have early-onset or more severe AGA, have a family history, or have other risk factors, measurement of waist circumference and blood pressure and consideration of fasting glucose or glycated hemoglobin and fasting lipid testing may be appropriate within a shared decision-making framework. Because hypertension and diabetes are risk factors for chronic kidney disease, the need for broader cardiometabolic and renal risk assessment should also be considered in patients with AGA and either condition. The triglyceride-to-HDL-C ratio and AIP may reflect additional metabolic or atherosclerotic risk; however, the included studies did not provide poolable binary prevalence data for these indices. The high heterogeneity observed in this review may reflect differences in region, age and sex distribution, hospital- versus community-based sampling, AGA diagnosis and grading, thresholds for MetS and its components, and methods used to ascertain comorbidities.

## 12 Conclusions

MetS and several metabolic abnormalities occur among patients with AGA; however, prevalence estimates vary substantially across studies. The pooled prevalence was 23.7% for MetS, 36.0% for abdominal obesity, 30.7% for hypertension or elevated blood pressure, 13.3% for diabetes mellitus, 24.5% for dyslipidemia, 23.6% for hypertriglyceridemia, and 28.8% for low HDL-C. Evidence for elevated LDL-C was based on only two small studies and remains uncertain. In clinical practice, cardiometabolic assessment should be individualized according to a patient’s overall risk profile. Future prospective studies with appropriate comparison groups and adequate adjustment for confounding are warranted.

## 13 Strengths and limitations

The main strength of this study was the restriction to cross-sectional and cohort studies and the exclusion of case-control studies, with numerators and denominators for each outcome verified against the original reports. We also reported REML random-effects estimates, I², τ², 95% PIs, leave-one-out analyses, and Egger’s test only when the number of studies was sufficient. The review covered MetS and multiple components and included exploratory analyses by diagnostic definition, region, sex, and AGA severity. Several limitations should be considered. Most evidence was cross-sectional and therefore could not establish temporality or causality. Geographic representation was uneven, and some subgroups contained only one or two studies. Definitions of MetS and its components, AGA grading systems, and sampling sources varied considerably. Some sex- and severity-specific strata were not independent. Heterogeneity was high, and PIs were wide for most outcomes. Data were sparse for elevated LDL-C, the triglyceride-to-HDL-C ratio, AIP, and uric acid. Restriction to full-text articles in Chinese or English may have introduced language bias, and unpublished studies may have been missed. Finally, only 11 studies were rated as high quality, whereas 15 were rated as moderate quality and two as low quality. Lack of assessor blinding, incomplete reporting of missing-data handling, and limited longitudinal follow-up were the most common methodological concerns, further limiting interpretation and generalizability. In particular, female-specific data are sparse, and the Chinese estimate does not capture the geographic diversity of the national population. Quality-stratified estimates were also confounded by region and sampling setting, and the low-quality stratum included only one study.
