# Supplementary material for: Prevalence of metabolic syndrome in patients with androgenetic alopecia: an analysis of current evidence
Source: Front Endocrinol (Lausanne). 2026 Sep 22;17:1904726. doi: 10.3389/fendo.2026.1904726 (PMC13639579; doi:10.3389/fendo.2026.1904726)
Supplement: Supplementary file 1 [file SupplementaryFile12.pdf]

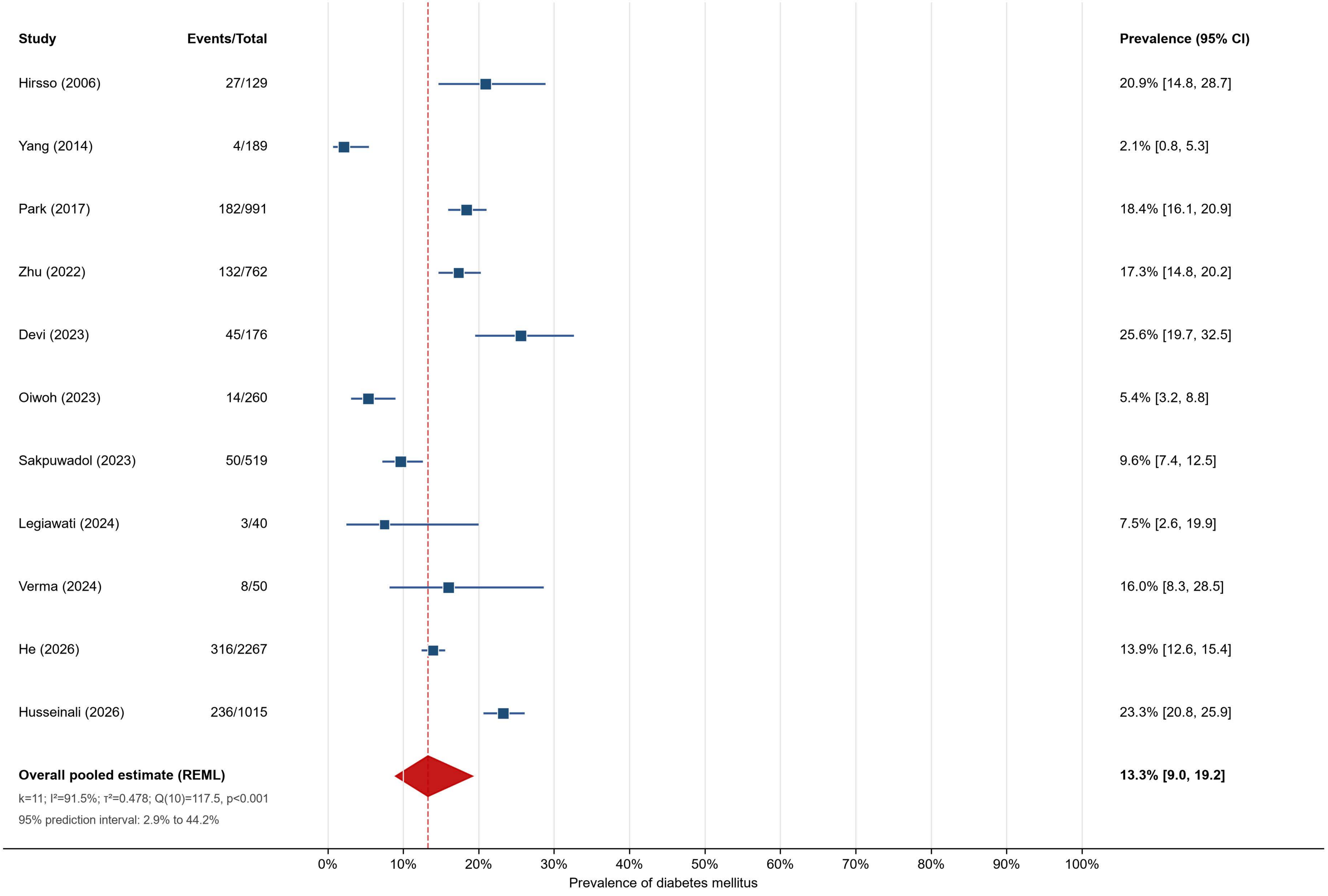
Study
Events/Total
Prevalence (95% CI)
Hirsso (2006) 27/129 20.9% [14.8, 28.7]
Yang (2014) 4/189 2.1% [0.8, 5.3]
Park (2017) 182/991 18.4% [16.1, 20.9]
Zhu (2022) 132/762 17.3% [14.8, 20.2]
Devi (2023) 45/176 25.6% [19.7, 32.5]
Oiwoh (2023) 14/260 5.4% [3.2, 8.8]
Sakpuwadol (2023) 50/519 9.6% [7.4, 12.5]
Legiawati (2024) 3/40 7.5% [2.6, 19.9]
Verma (2024) 8/50 16.0% [8.3, 28.5]
He (2026) 316/2267 13.9% [12.6, 15.4]
Husseinali (2026) 236/1015 23.3% [20.8, 25.9]
Overall pooled estimate (REML) 13.3% [9.0, 19.2]
k=11; I²=91.5%; τ²=0.478; Q(10)=117.5, p<0.001
95% prediction interval: 2.9% to 44.2%
0% 10% 20% 30% 40% 50% 60% 70% 80% 90% 100%
Prevalence of diabetes mellitus

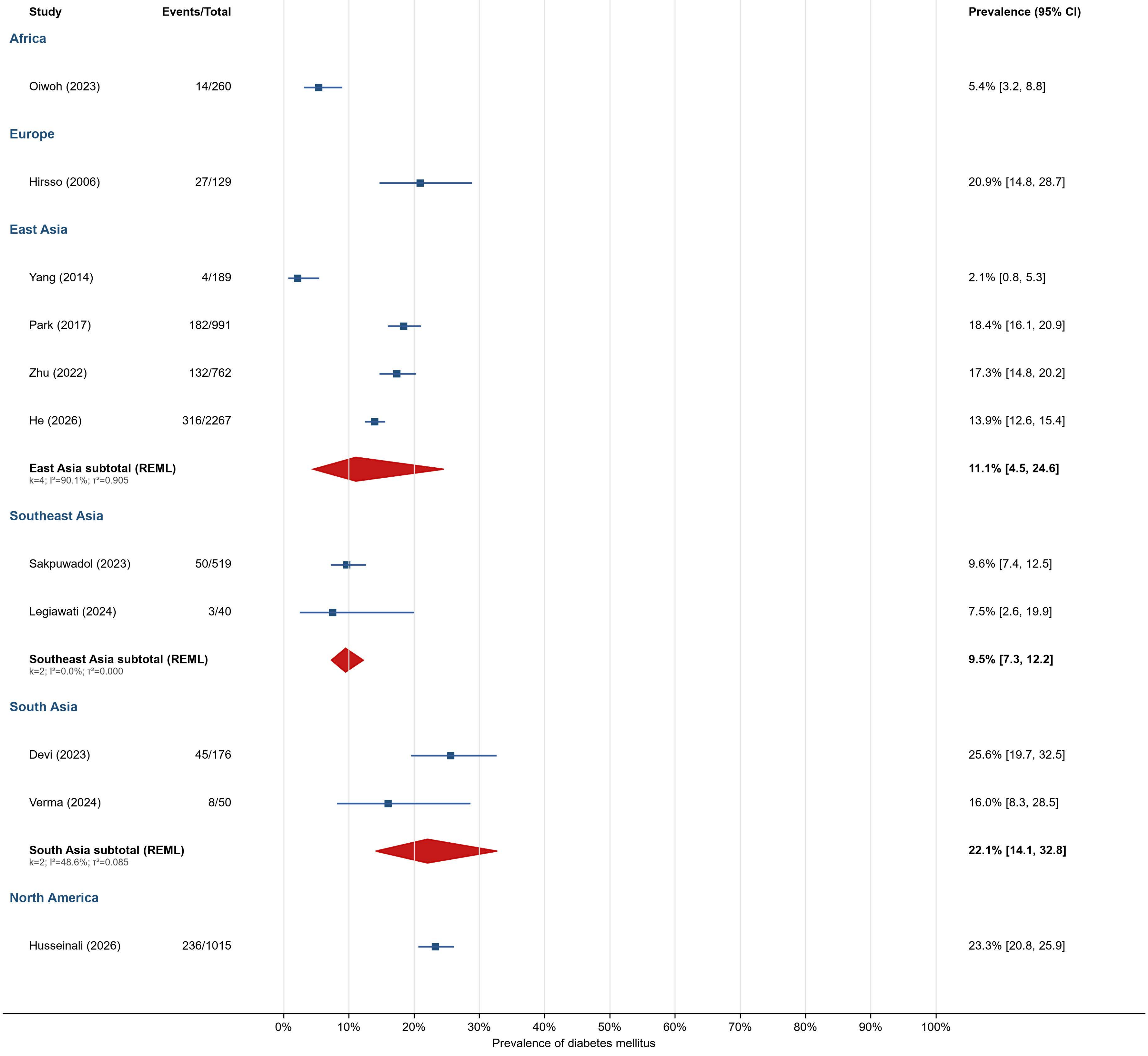
Study
Events/Total
Prevalence (95% CI)
Africa
Oiwoh (2023)
14/260
5.4% [3.2, 8.8]
Europe
Hirsso (2006)
27/129
20.9% [14.8, 28.7]
East Asia
Yang (2014)
4/189
2.1% [0.8, 5.3]
Park (2017)
182/991
18.4% [16.1, 20.9]
Zhu (2022)
132/762
17.3% [14.8, 20.2]
He (2026)
316/2267
13.9% [12.6, 15.4]
East Asia subtotal (REML)
k=4; I²=90.1%; τ²=0.905
11.1% [4.5, 24.6]
Southeast Asia
Sakpuwadol (2023)
50/519
9.6% [7.4, 12.5]
Legiawati (2024)
3/40
7.5% [2.6, 19.9]
Southeast Asia subtotal (REML)
k=2; I²=0.0%; τ²=0.000
9.5% [7.3, 12.2]
South Asia
Devi (2023)
45/176
25.6% [19.7, 32.5]
Verma (2024)
8/50
16.0% [8.3, 28.5]
South Asia subtotal (REML)
k=2; I²=48.6%; τ²=0.085
22.1% [14.1, 32.8]
North America
Husseinali (2026)
236/1015
23.3% [20.8, 25.9]
0%
10%
20%
30%
40%
50%
60%
70%
80%
90%
100%
Prevalence of diabetes mellitus

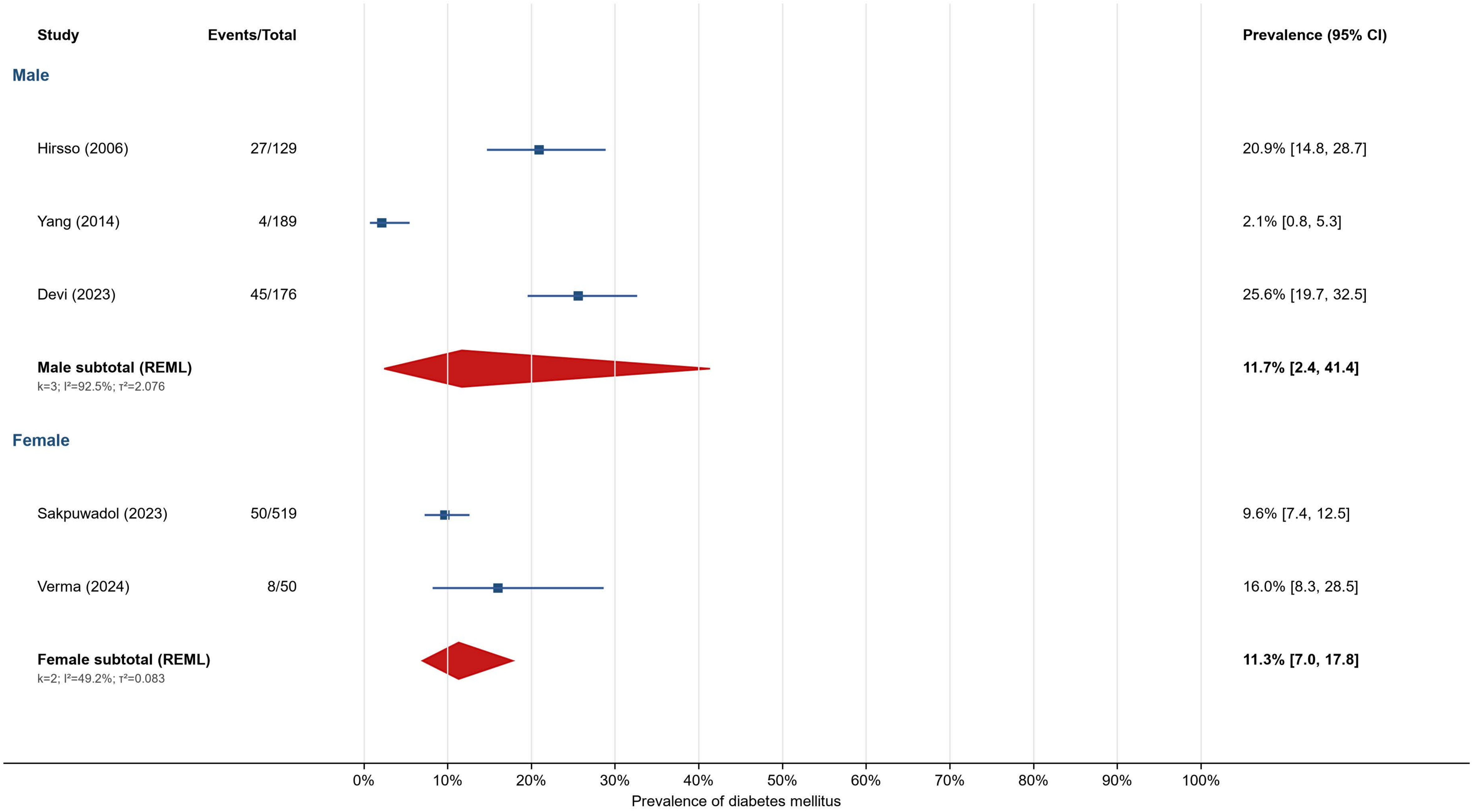

Study
Events/Total
Prevalence (95% CI)
Male
Hirsso (2006)
27/129
20.9% [14.8, 28.7]
Yang (2014)
4/189
2.1% [0.8, 5.3]
Devi (2023)
45/176
25.6% [19.7, 32.5]
Male subtotal (REML)
k=3; I²=92.5%; τ²=2.076
11.7% [2.4, 41.4]
Female
Sakpuwadol (2023)
50/519
9.6% [7.4, 12.5]
Verma (2024)
8/50
16.0% [8.3, 28.5]
Female subtotal (REML)
k=2; I²=49.2%; τ²=0.083
11.3% [7.0, 17.8]
0%
10%
20%
30%
40%
50%
60%
70%
80%
90%
100%
Prevalence of diabetes mellitus
