# Supplementary material for: Prevalence of metabolic syndrome in patients with androgenetic alopecia: an analysis of current evidence
Source: Front Endocrinol (Lausanne). 2026 Sep 22;17:1904726. doi: 10.3389/fendo.2026.1904726 (PMC13639579; doi:10.3389/fendo.2026.1904726)
Supplement: Supplementary file 2 [file SupplementaryFile13.pdf]

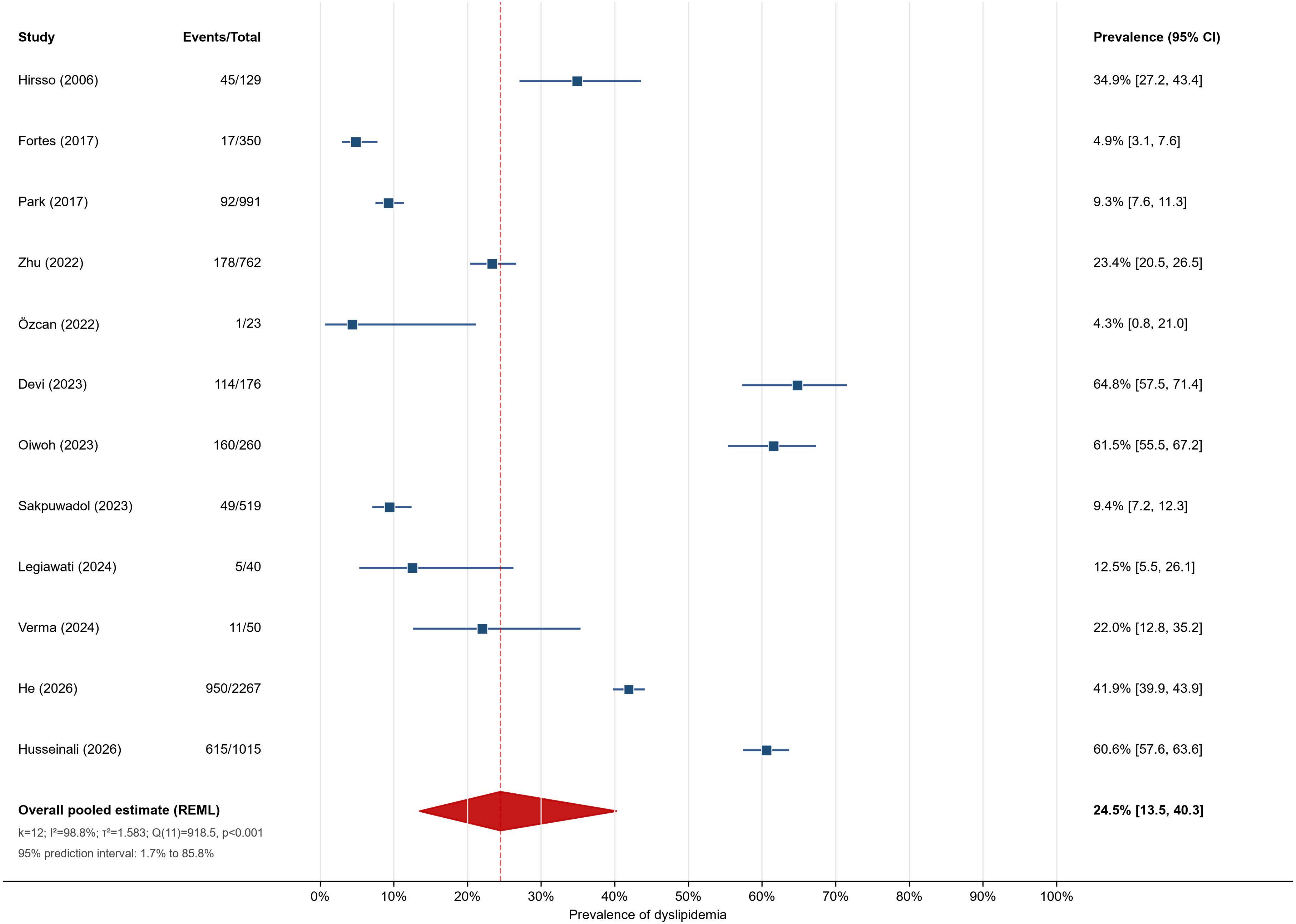
Study
Events/Total
Prevalence (95% CI)
Hirsso (2006)
45/129
34.9% [27.2, 43.4]
Fortes (2017)
17/350
4.9% [3.1, 7.6]
Park (2017)
92/991
9.3% [7.6, 11.3]
Zhu (2022)
178/762
23.4% [20.5, 26.5]
Özcan (2022)
1/23
4.3% [0.8, 21.0]
Devi (2023)
114/176
64.8% [57.5, 71.4]
Oiwoh (2023)
160/260
61.5% [55.5, 67.2]
Sakpuwadol (2023)
49/519
9.4% [7.2, 12.3]
Legiawati (2024)
5/40
12.5% [5.5, 26.1]
Verma (2024)
11/50
22.0% [12.8, 35.2]
He (2026)
950/2267
41.9% [39.9, 43.9]
Husseinali (2026)
615/1015
60.6% [57.6, 63.6]
Overall pooled estimate (REML)
24.5% [13.5, 40.3]
k=12; I²=98.8%; τ²=1.583; Q(11)=918.5, p<0.001
95% prediction interval: 1.7% to 85.8%
0%
10%
20%
30%
40%
50%
60%
70%
80%
90%
100%
Prevalence of dyslipidemia

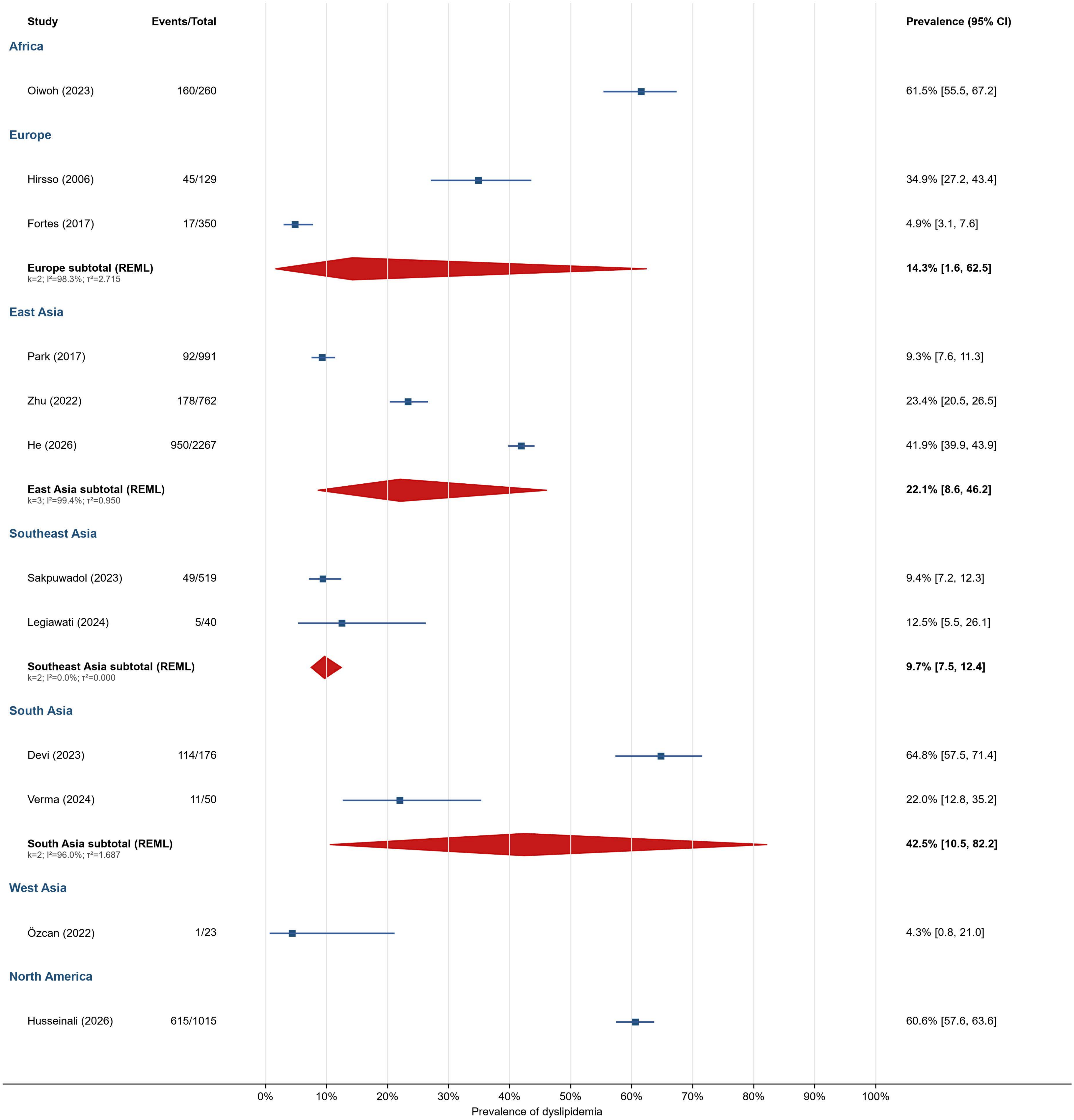
Study
Events/Total
Prevalence (95% CI)
Africa
Oiwoh (2023)
160/260
61.5% [55.5, 67.2]
Europe
Hirsso (2006)
45/129
34.9% [27.2, 43.4]
Fortes (2017)
17/350
4.9% [3.1, 7.6]
Europe subtotal (REML)
k=2; I²=98.3%; τ²=2.715
14.3% [1.6, 62.5]
East Asia
Park (2017)
92/991
9.3% [7.6, 11.3]
Zhu (2022)
178/762
23.4% [20.5, 26.5]
He (2026)
950/2267
41.9% [39.9, 43.9]
East Asia subtotal (REML)
k=3; I²=99.4%; τ²=0.950
22.1% [8.6, 46.2]
Southeast Asia
Sakpuwadol (2023)
49/519
9.4% [7.2, 12.3]
Legiawati (2024)
5/40
12.5% [5.5, 26.1]
Southeast Asia subtotal (REML)
k=2; I²=0.0%; τ²=0.000
9.7% [7.5, 12.4]
South Asia
Devi (2023)
114/176
64.8% [57.5, 71.4]
Verma (2024)
11/50
22.0% [12.8, 35.2]
South Asia subtotal (REML)
k=2; I²=96.0%; τ²=1.687
42.5% [10.5, 82.2]
West Asia
Özcan (2022)
1/23
4.3% [0.8, 21.0]
North America
Husseinali (2026)
615/1015
60.6% [57.6, 63.6]
0%
10%
20%
30%
40%
50%
60%
70%
80%
90%
100%
Prevalence of dyslipidemia

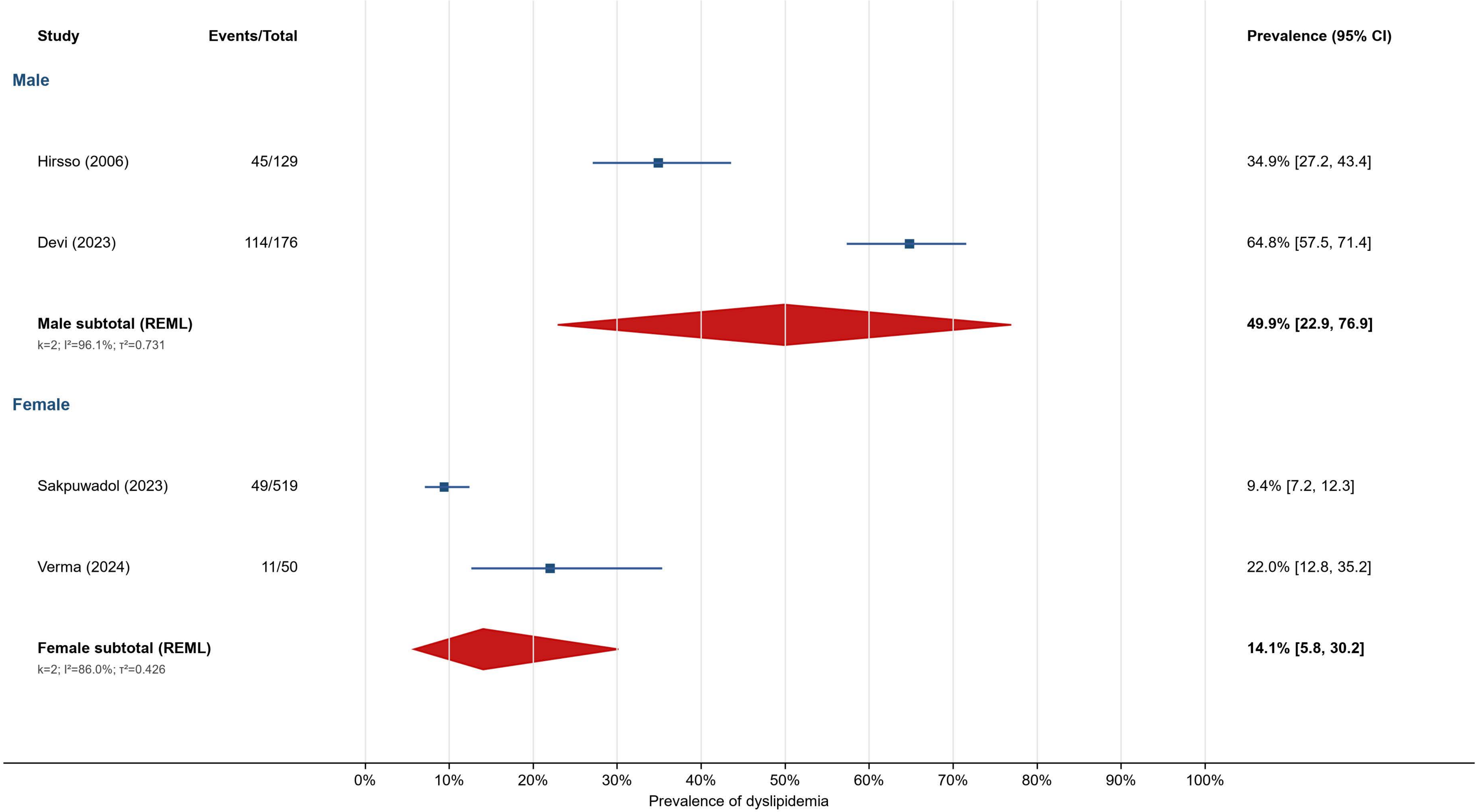
Study
Events/Total
Prevalence (95% CI)
Male
Hirsso (2006)
45/129
34.9% [27.2, 43.4]
Devi (2023)
114/176
64.8% [57.5, 71.4]
Male subtotal (REML)
k=2; I²=96.1%; τ²=0.731
49.9% [22.9, 76.9]
Female
Sakpuwadol (2023)
49/519
9.4% [7.2, 12.3]
Verma (2024)
11/50
22.0% [12.8, 35.2]
Female subtotal (REML)
k=2; I²=86.0%; τ²=0.426
14.1% [5.8, 30.2]
0%
10%
20%
30%
40%
50%
60%
70%
80%
90%
100%
Prevalence of dyslipidemia

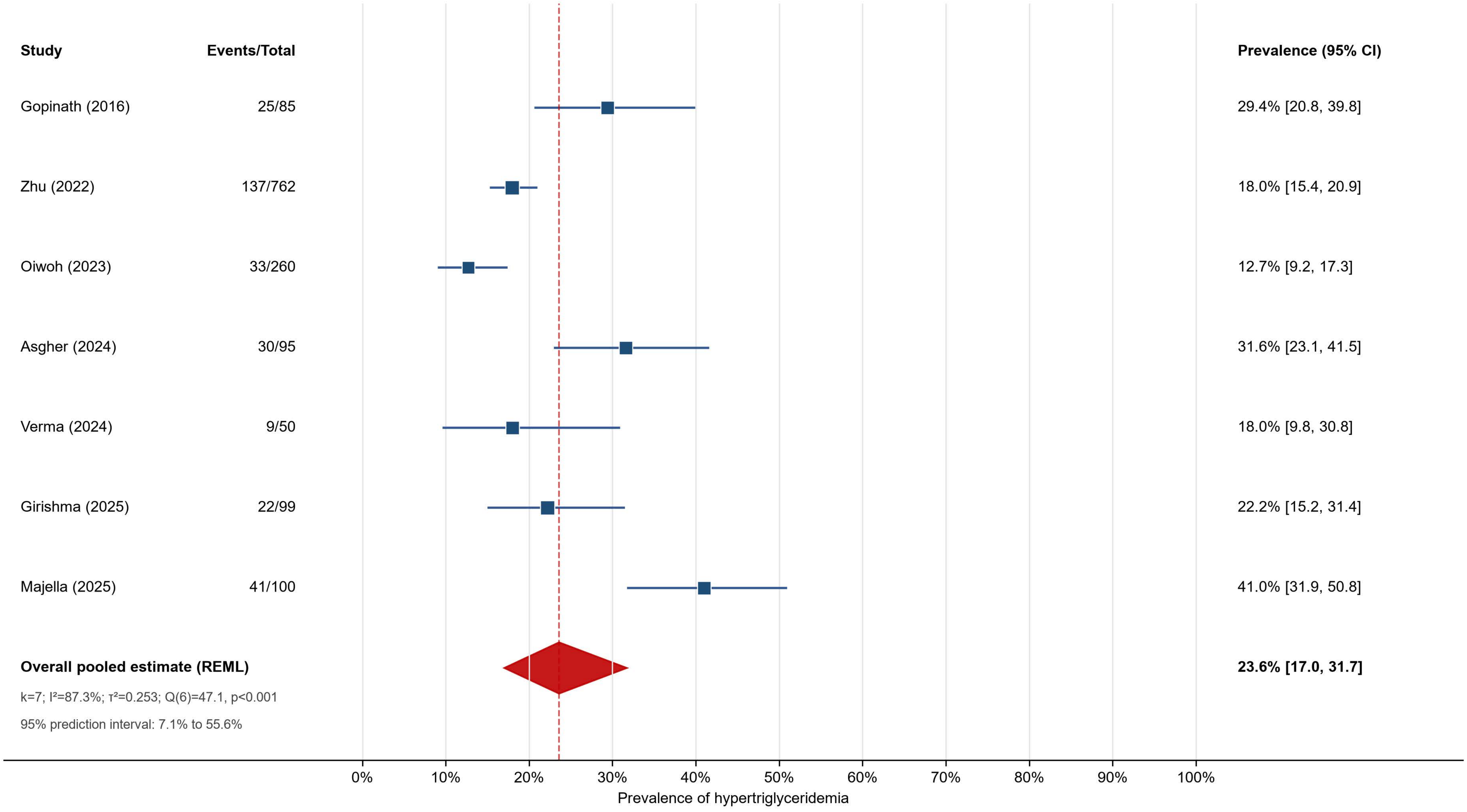
Study
Events/Total
Prevalence (95% CI)
Gopinath (2016)
25/85
29.4% [20.8, 39.8]
Zhu (2022)
137/762
18.0% [15.4, 20.9]
Oiwoh (2023)
33/260
12.7% [9.2, 17.3]
Asgher (2024)
30/95
31.6% [23.1, 41.5]
Verma (2024)
9/50
18.0% [9.8, 30.8]
Girishma (2025)
22/99
22.2% [15.2, 31.4]
Majella (2025)
41/100
41.0% [31.9, 50.8]
Overall pooled estimate (REML)
23.6% [17.0, 31.7]
k=7; I²=87.3%; τ²=0.253; Q(6)=47.1, p<0.001
95% prediction interval: 7.1% to 55.6%
0%
10%
20%
30%
40%
50%
60%
70%
80%
90%
100%
Prevalence of hypertriglyceridemia

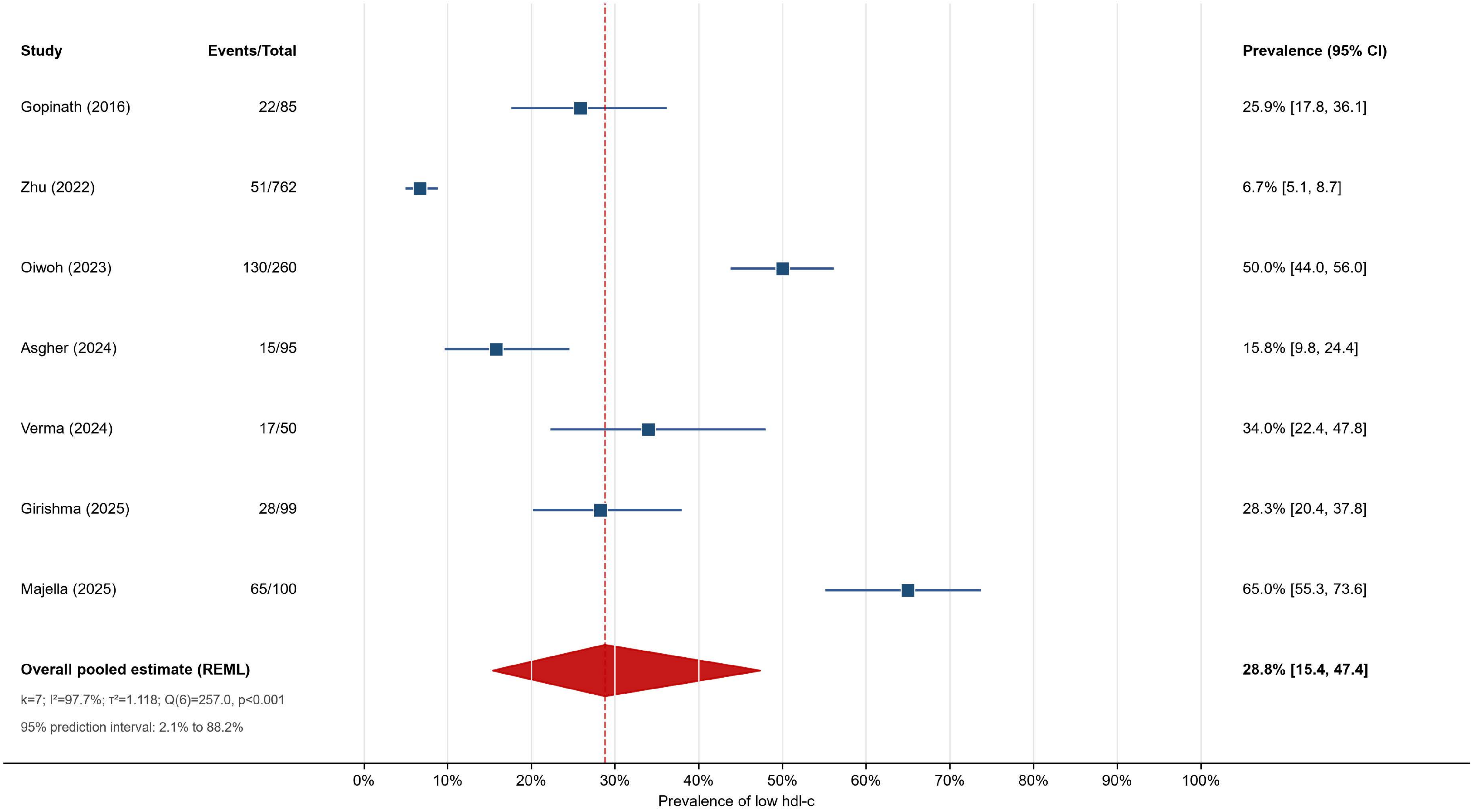

Study
Events/Total
Prevalence (95% CI)
Gopinath (2016)
22/85
25.9% [17.8, 36.1]
Zhu (2022)
51/762
6.7% [5.1, 8.7]
Oiwoh (2023)
130/260
50.0% [44.0, 56.0]
Asgher (2024)
15/95
15.8% [9.8, 24.4]
Verma (2024)
17/50
34.0% [22.4, 47.8]
Girishma (2025)
28/99
28.3% [20.4, 37.8]
Majella (2025)
65/100
65.0% [55.3, 73.6]
Overall pooled estimate (REML)
28.8% [15.4, 47.4]
k=7; I²=97.7%; τ²=1.118; Q(6)=257.0, p<0.001
95% prediction interval: 2.1% to 88.2%
0%
10%
20%
30%
40%
50%
60%
70%
80%
90%
100%
Prevalence of low hdl-c

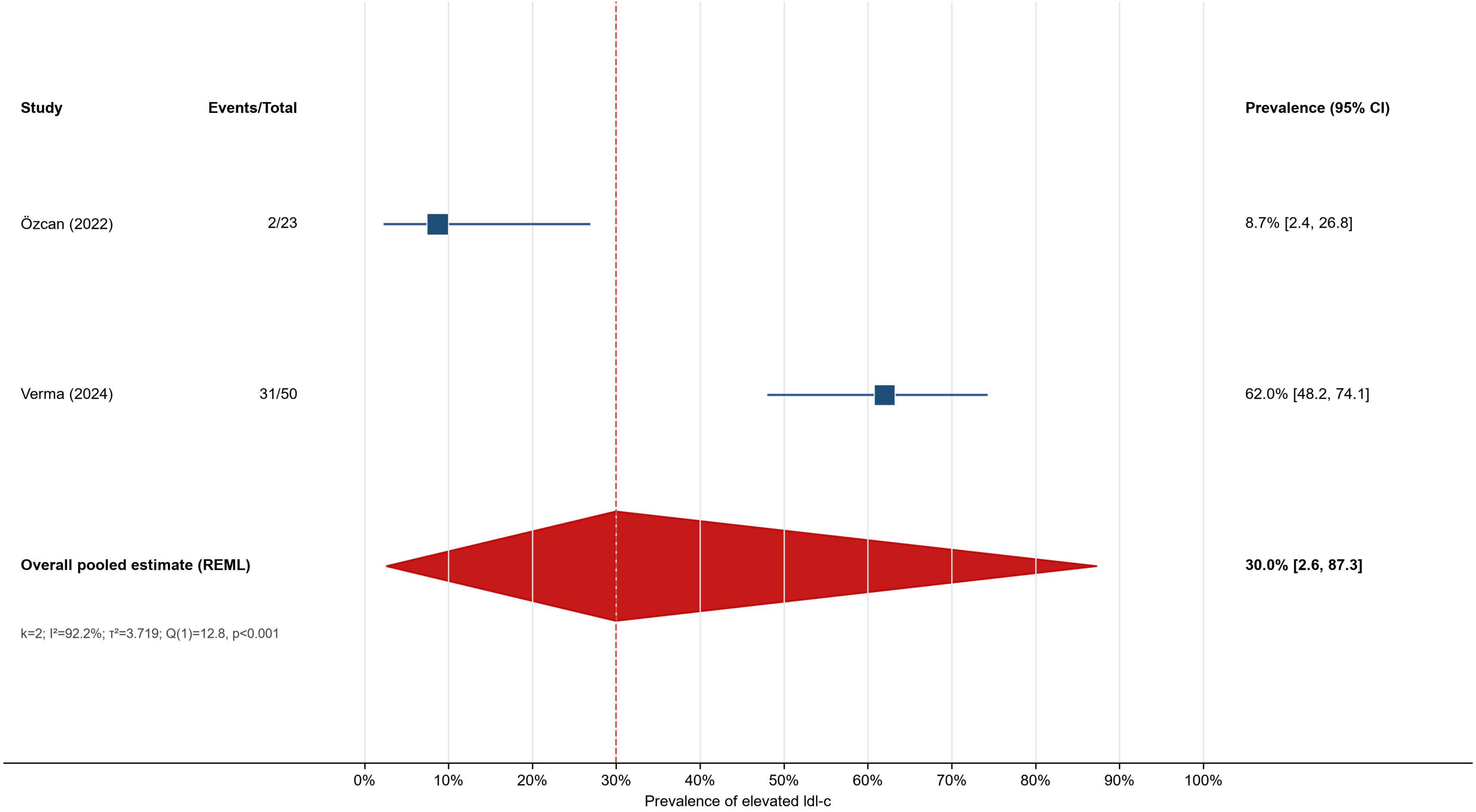
Study
Events/Total
Prevalence (95% CI)
Özcan (2022)
2/23
8.7% [2.4, 26.8]
Verma (2024)
31/50
62.0% [48.2, 74.1]
Overall pooled estimate (REML)
30.0% [2.6, 87.3]
k=2; I²=92.2%; τ²=3.719; Q(1)=12.8, p<0.001
0%
10%
20%
30%
40%
50%
60%
70%
80%
90%
100%
Prevalence of elevated ldl-c
