# Supplementary material for: Prevalence of metabolic syndrome in patients with androgenetic alopecia: an analysis of current evidence
Source: Front Endocrinol (Lausanne). 2026 Sep 22;17:1904726. doi: 10.3389/fendo.2026.1904726 (PMC13639579; doi:10.3389/fendo.2026.1904726)
Supplement: Supplementary file 3 [file SupplementaryFile14.pdf]

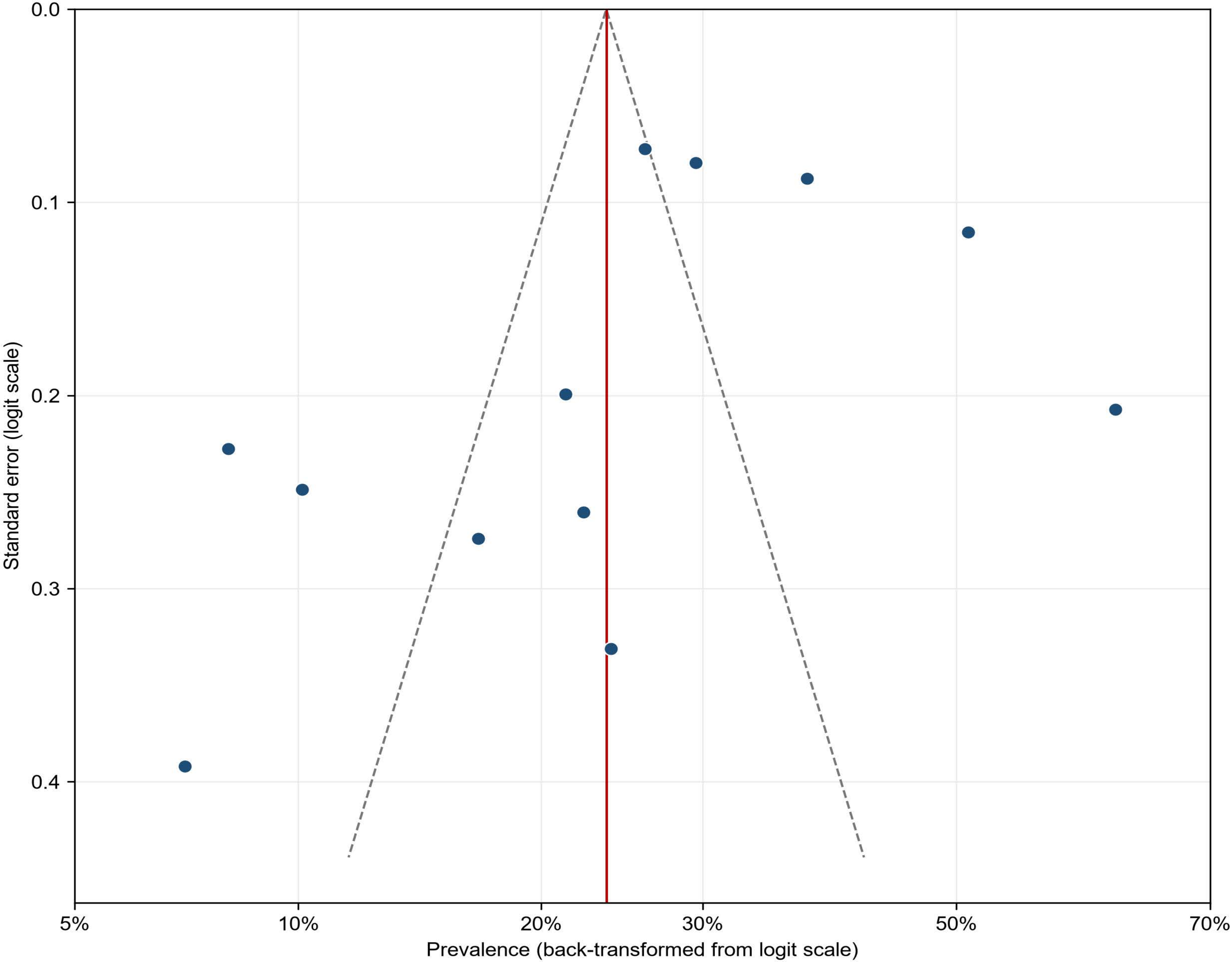

Supplementary Figure 15. Funnel plot of the prevalence of MetS in AGA patients

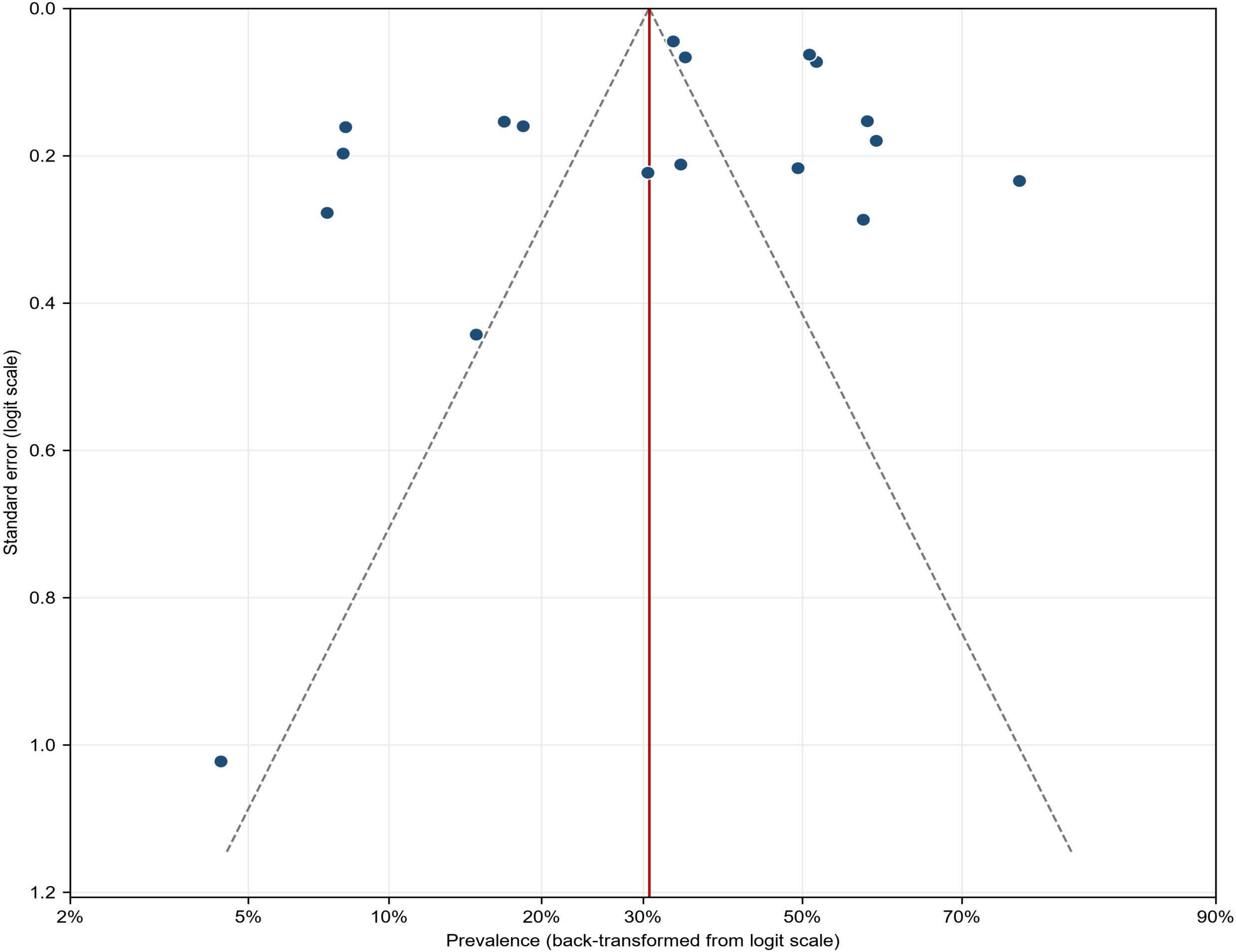

Supplementary Figure 16. Funnel plot of the prevalence of hypertension in AGA patients

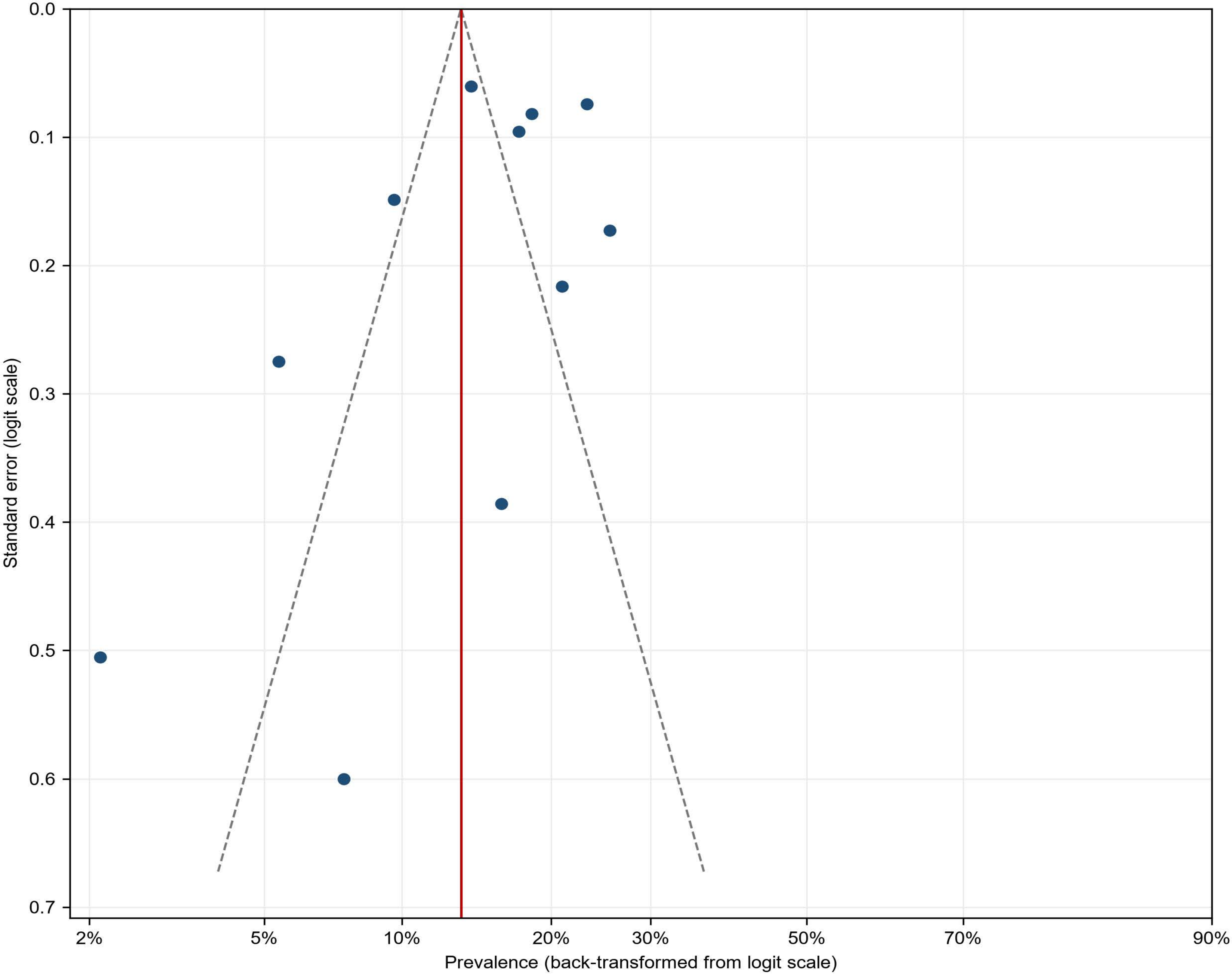

Supplementary Figure 17. Funnel plot of the prevalence of diabetes in AGA patients

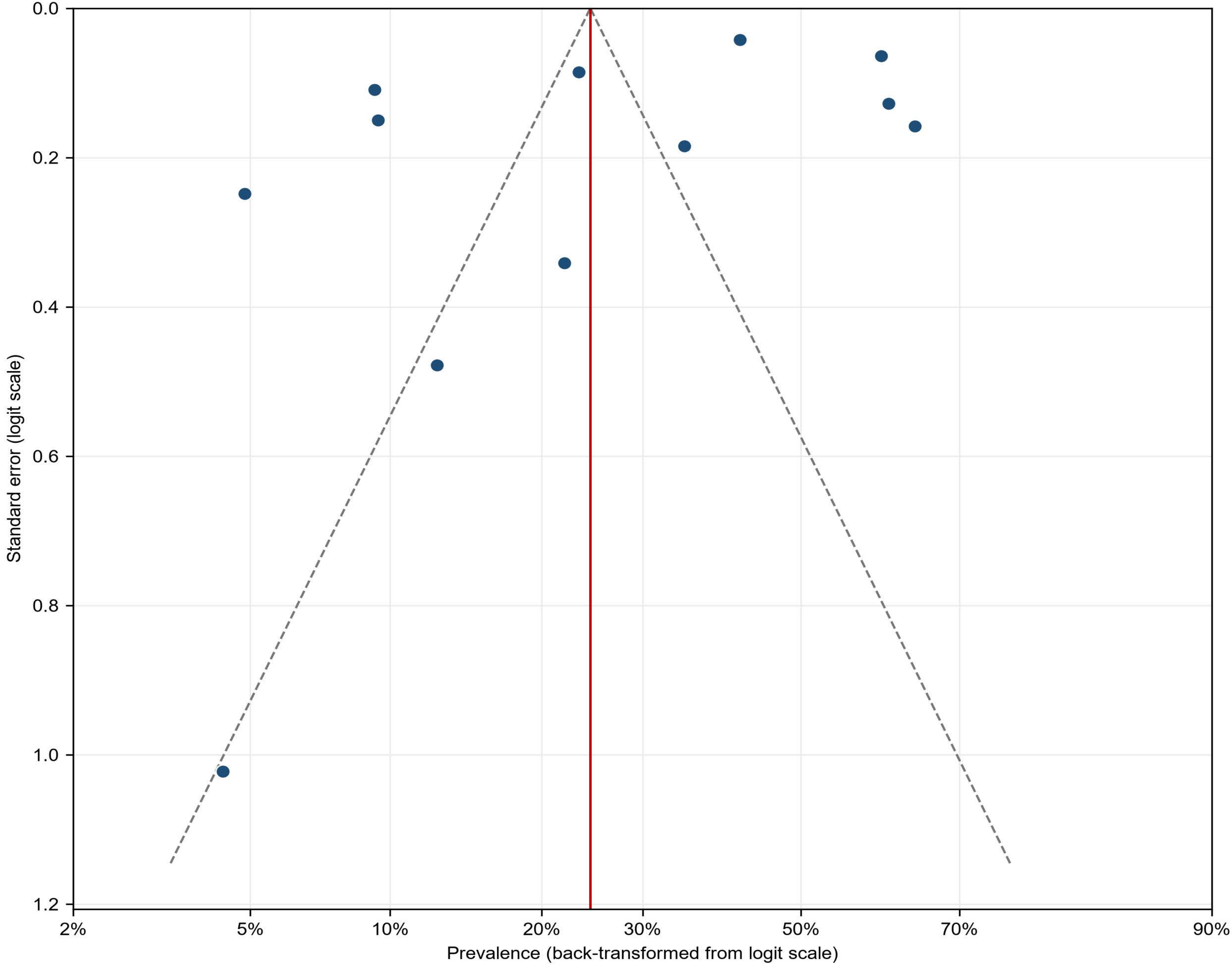

Supplementary Figure 18. Funnel plot of the prevalence of dyslipidemia in AGA patients
