# Supplementary material for: Prevalence of metabolic syndrome in patients with androgenetic alopecia: an analysis of current evidence
Source: Front Endocrinol (Lausanne). 2026 Sep 22;17:1904726. doi: 10.3389/fendo.2026.1904726 (PMC13639579; doi:10.3389/fendo.2026.1904726)
Supplement: Supplementary file 5 [file Table2.docx]

**Table S2.** Search strategy.

| **Platform** | **Search strategy** |
| --- | --- |
| **Pubmed** | (("Alopecia"[Mesh]) OR ((((((((((((((Androgenetic Alopecia[Title/Abstract]) OR (Alopecia, Androgenetic[Title/Abstract])) OR (Androgenic Alopecia[Title/Abstract])) OR (Alopecia, Androgenic[Title/Abstract])) OR (Alopecias, Androgenic[Title/Abstract])) OR (Androgenic Alopecias[Title/Abstract])) OR (Female Pattern Baldness[Title/Abstract])) OR (Baldness, Female Pattern[Title/Abstract])) OR (Alopecia, Male Pattern[Title/Abstract])) OR (Male Pattern Alopecia[Title/Abstract])) OR (Male Pattern Baldness[Title/Abstract])) OR (Baldness, Male Pattern[Title/Abstract])) OR (alopecia seborrhoeica[Title/Abstract])) OR (Seborrheic Alopecia[Title/Abstract]))) AND (("Cross-Sectional Studies"[Mesh]) OR (((((((((((((((((Studies, Cross-Sectional[Title/Abstract]) OR (Study, Cross-Sectional[Title/Abstract])) OR (Cross-Sectional Survey[Title/Abstract])) OR (Cross-Sectional Surveys[Title/Abstract])) OR (Survey, Cross-Sectional[Title/Abstract])) OR (Surveys, Cross-Sectional[Title/Abstract])) OR (Surveys, Disease Frequency[Title/Abstract])) OR (Disease Frequency Survey[Title/Abstract])) OR (Survey, Disease Frequency[Title/Abstract])) OR (Disease Frequency Surveys[Title/Abstract])) OR (Analysis, Cross-Sectional[Title/Abstract])) OR (Analysis, Cross Sectional[Title/Abstract])) OR (Cross-Sectional Analysis[Title/Abstract])) OR (Analyses, Cross-Sectional[Title/Abstract])) OR (Cross-Sectional Analyses[Title/Abstract])) OR (Prevalence[Title/Abstract])) OR (Epidemiology[Title/Abstract]))) |
| **Web of Science** | TS=("Androgenetic Alopecia" OR "Alopecia, Androgenetic" OR "Androgenic Alopecia" OR "Alopecia, Androgenic" OR "Alopecias, Androgenic" OR "Androgenic Alopecias" OR "Female Pattern Baldness" OR "Baldness, Female Pattern" OR "Alopecia, Male Pattern" OR "Male Pattern Alopecia" OR "Male Pattern Baldness" OR "Baldness, Male Pattern" OR "alopecia seborrhoeica") AND TS=("Cross Sectional Studies" OR "Cross-Sectional Study" OR "Studies, Cross-Sectional" OR "Study, Cross-Sectional" OR "Cross-Sectional Survey" OR "Cross Sectional Survey" OR "Cross-Sectional Surveys" OR "Survey, Cross-Sectional" OR "Surveys, Cross-Sectional" OR "Surveys, Disease Frequency" OR "Disease Frequency Survey" OR "Survey, Disease Frequency" OR "Disease Frequency Surveys" OR "Analysis, Cross-Sectional" OR "Analyses, Cross-Sectional" OR "Analysis, Cross Sectional" OR "Cross-Sectional Analyses" OR "Cross-Sectional Analysis" OR "Cross Sectional Analysis" OR "Analyses, Cross Sectional" OR "Cross Sectional Analyses" OR "Prevalence Studies" OR "Prevalence Study" OR "Studies, Prevalence" OR "Study, Prevalence") |
| **Cochrane** | ("Androgenetic Alopecia" OR "Alopecia, Androgenetic" OR "Androgenic Alopecia" OR "Alopecia, Androgenic" OR "Alopecias, Androgenic" OR "Androgenic Alopecias" OR "Female Pattern Baldness" OR "Baldness, Female Pattern" OR "Alopecia, Male Pattern" OR "Male Pattern Alopecia" OR "Male Pattern Baldness" OR "Baldness, Male Pattern" OR alopecia seborrhoeica) AND ("Cross Sectional Studies" OR "Cross-Sectional Study" OR "Studies, Cross-Sectional" OR "Study, Cross-Sectional" OR "Cross-Sectional Survey" OR "Cross Sectional Survey" OR "Cross-Sectional Surveys" OR "Survey, Cross-Sectional" OR "Surveys, Cross-Sectional" OR "Surveys, Disease Frequency" OR "Disease Frequency Survey" OR "Survey, Disease Frequency" OR "Disease Frequency Surveys" OR "Analysis, Cross-Sectional" OR "Analyses, Cross-Sectional" OR "Analysis, Cross Sectional" OR "Cross-Sectional Analyses" OR "Cross-Sectional Analysis" OR "Cross Sectional Analysis" OR "Analyses, Cross Sectional" OR "Cross Sectional Analyses" OR "Prevalence Studies" OR "Prevalence Study" OR "Studies, Prevalence" OR "Study, Prevalence") |

Continued

| **Platform** | **Search strategy** |
| --- | --- |
| **Embase** | TS=(Androgenetic Alopecia OR “Alopecia, Androgenetic” OR Androgenic Alopecia OR “Alopecia, Androgenic” OR “Alopecias, Androgenic” OR Androgenic Alopecias OR Female Pattern Baldness OR “Baldness, Female Pattern” OR “Alopecia, Male Pattern” OR Male Pattern Alopecia OR Male Pattern Baldness OR “Baldness, Male Pattern” OR alopecia seborrhoeica ) AND TS=(Cross Sectional Studies OR Cross-Sectional Study OR “Studies, Cross-Sectional” OR “Study, Cross-Sectional” OR Cross-Sectional Survey OR Cross Sectional Survey OR Cross-Sectional Surveys OR “Survey, Cross-Sectional” OR “Surveys, Cross-Sectional” OR “Surveys, Disease Frequency” OR Disease Frequency Survey OR “Survey, Disease Frequency” OR Disease Frequency Surveys OR “Analysis, Cross-Sectional” OR “Analyses, Cross-Sectional” OR “Analysis, Cross Sectional” OR Cross-Sectional Analyses OR Cross-Sectional Analysis OR Cross Sectional Analysis OR “Analyses, Cross Sectional” OR Cross Sectional Analyses OR Prevalence Studies OR Prevalence Study OR “Studies, Prevalence” OR “Study, Prevalence” ) |
| **CNKI** | 雄激素性脱发+ 脂溢性脱发 + 女性型脱发 + 男性型脱发 And 横断面研究+问卷调查+问卷+流行病学+横断面 |
| **Wangfang and VIP** | 雄激素性脱发 or 脂溢性脱发 or 女性型脱发 or 男性型脱发 And 横断面研究 or 问卷调查 or 问卷 or 流行病学 or 横断面 |
